# Retention Time Trajectory Matching for Peak Identification in Chromatographic Analysis

**DOI:** 10.3390/s23136029

**Published:** 2023-06-29

**Authors:** Wenzhe Zang, Ruchi Sharma, Maxwell Wei-Hao Li, Xudong Fan

**Affiliations:** 1 Department of Biomedical Engineering, University of Michigan, 1101 Beal Avenue, Ann Arbor, MI 48109, USA; 2 Center for Wireless Integrated MicroSensing and Systems (WIMS2), University of Michigan, Ann Arbor, MI 48109, USA; 3 Max Harry Weil Institute for Critical Care Research and Innovation, University of Michigan, Ann Arbor, MI 48109, USA; 4 Department of Electrical Engineering and Computer Science, University of Michigan, Ann Arbor, MI 48109, USA

**Keywords:** gas chromatogram, liquid chromatography, retention time, retention time drift, peak identification

## Abstract

Retention time drift caused by fluctuations in physical factors such as temperature ramping rate and carrier gas flow rate is ubiquitous in chromatographic measurements. Proper peak matching and identification across different chromatograms is critical prior to any subsequent analysis but is challenging without using mass spectrometry. The purpose of this work was to describe and validate a peak matching and identification method called retention time trajectory (RTT) matching that can be used in targeted analyses free of mass spectrometry. This method uses chromatographic retention times as the only input and identifies peaks associated with any subset of a predefined set of target compounds. An RTT is a two-dimensional (2D) curve formed uniquely by the retention times of the chromatographic peaks. The RTTs obtained from the chromatogram of a sample under test and those pre-installed in a library are matched and statistically compared. The best matched pair implies identification. Unlike most existing peak-alignment methods, no mathematical warping or transformation is involved. Based on the experimentally characterized RTT, an RTT hybridization method was also developed to rapidly generate more RTTs and expand the library without performing actual time-consuming chromatographic measurements, which enables successful peak matching even for chromatograms with severe retention time drifts. Additionally, 3.15 × 10^5^ tests using experimentally obtained gas chromatograms and 2 × 10^12^ tests using two publicly available fruit metabolomics datasets validated the proposed method, demonstrating real-time peak/interferent identification.

## 1. Introduction

Gas chromatography (GC)-based volatile organic compound (VOC) analysis can be classified into untargeted analysis and targeted analysis. The former involves evaluation of chemical substances in an unknown sample, whereas the latter aims only at a predetermined list of interesting compounds or a subset of those, with all other VOCs treated as interferents. Due to the complexity of sample composition and the lack of pre-existing knowledge, accurate identification in untargeted analysis requires confirmation or cross-validation by at least two parameters, such as chromatographic retention time (RT) and mass spectrometry (MS) fragmentation profile. In contrast, in targeted analysis, oftentimes only the retention time is used for compound identification in order to avoid using bulky and expensive mass spectrometry. Therefore, targeted analysis has broad applications in on-site real-time measurements, such as environmental protection [1,2,3], workplace environment monitoring [4,5,6,7,8], industries (e.g., petroleum [9,10,11] and food [12,13]), and metabolomics [14,15,16].

For targeted analysis, the retention time of each peak in the GC chromatogram is compared with the pre-installed values of all compounds of interest in a library. In any given sample, a positive alarm is reported when the retention time of a peak matches a corresponding time in the library; the lack of any match instead means that a peak would be ignored or reported as an interferent. However, variations in physical factors such as ambient temperature, column temperature ramping profile, and carrier gas flow rate can affect the retention time of each peak from run to run, which hinders identification or triggers false alarms. The inability to correctly identify peaks with only retention times is exacerbated when a sample contains a large number of compounds or when some of the targeted peaks are closely eluted out in a chromatogram. Consequently, proper matching or alignment of chromatographic peaks across different samples is a crucial preprocessing step that must be performed prior to any subsequent analysis.

A simple and popular solution is data binning, which divides the signals into bins (e.g., histograms) and incorporates all data into a recognition profile for each measurement [17,18]. The binning method is easy to use and shows acceptable performance in processing both chromatograms and spectra when the peak drift from sample to sample is much smaller than the distance between two adjacent peaks. However, in the presence of large peak drifts, this approach suffers from reduced resolution and information loss (see illustrations in Figure S1). The time warping technique, such as segment-wise correlation optimized warping (COW) [19], point-wise dynamic time warping (DTW) [20], global polynomial model-based parametric time warping (PTW) [21,22], multiscale peak alignment (MSPA) [23], and other variants [24,25,26,27], is one of most commonly adopted methods to correct retention time drifts across chromatograms. It aligns a whole measured chromatogram profile against a reference chromatogram using pattern recognition routines in order to achieve peak identification. While time warping is powerful and works well with samples of various complexities, an accurate warping-based aligning demands the fine tuning of alignment parameters, which can often involve human intervention, thus making automated peak identification less reliable. Moreover, all warping-based methods can suffer, to different degrees, from misalignments and are concentration-sensitive, even with samples of the same compositions. In some cases, warping-based aligning approaches may not be able to yield exactly the same retention time value for the same analyte from different measurements. Consequently, subsequent retention-time-based peak identification or statistical analysis often requires further value correction via data binning or clustering. Machine learning-based aligning approaches, which utilize artificial intelligence systems to acquire knowledge by extracting patterns from data, are also able to achieve positive alignment with decent accuracy [28,29,30,31,32], and they are amenable to automation without relying on human intervention. However, these approaches often employ the mass spectra of the peaks as one the of key subnetworks among the overall network architecture, making it more suitable for bulky mass-spectrometry-based analysis rather than for onsite monitoring where mass spectrometry is not used. Moreover, similar to most other machine learning-based approaches, machine learning-based aligning suffers from high computational cost during parameter training and feature extraction.

While the aforementioned methods can mitigate the peak drift issue across chromatograms, they all suffer from a number of drawbacks that make them unsuitable for compound identification in targeted analysis. First and foremost, the total number of peaks in the chromatogram obtained from the sample under test needs to be exactly the same as in the reference chromatogram. If only a subset or, in an extreme case, a single species of the compounds of interest (target compounds) is present in the sample under test, which is often the case for targeted analysis (e.g., pipeline leakage detection in a chemical plant), chromatogram aligning fails, as the detected peaks in the chromatogram have nothing to align with. Second, foreign interferents cannot be filtered out. If an additional peak is present in a chromatogram of the sample under test, it would be treated as one of the target compounds (misalignment) and/or it may cause failure in alignment of the whole chromatogram profile. Consequently, there has been an unmet need for an MS-free and chromatogram-based peak identification method that is able to identify any arbitrary subset of the target compounds as well as to report interferents.

**Figure 1:**
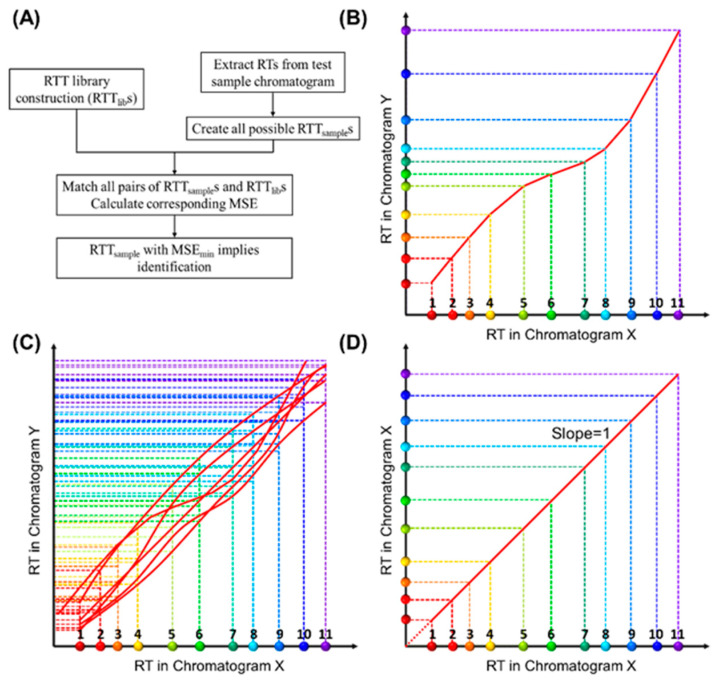
Conceptual illustration of retention time trajectory (RTT) and corresponding library construction. (**A**) Algorithm flow chart. (**B**) RTT of a chromatogram (Chromatogram Y)—RTT_Y_. The *X*-axis represents the retention time (RT_X_) from one of the chromatograms, which we call Chromatogram X. The colored dots along the *X*-axis represent different compounds and are numerically labelled as 1, 2, 3, …, 11. Similarly, the *Y*-axis represents the retention time, RT_Y_, in another chromatogram, Chromatogram Y. The entire set of coordinates, (RT_X,compound *i*_, RT_Y,compound *i*_), where “compound *i*” refers to a specific compound, forms a trajectory (red curve) in 2D. (**C**) Conceptual illustration of the RTT_lib_ library, which is composed of multiple chromatograms obtained under various experimental conditions (column temperature ramping profiles, carrier gas flow rate, etc.) using a mixture containing *all* target compounds (and internal standards if needed). (**D**) RTT of Chromatogram X (RTT_X_), where the retention time values along the *Y*-axis exactly match those along the *X*-axis (i.e., the slope of RTT_X_ is unity). Note that the RTT method, as shown in detail later, uses only the discrete peak retention time values. Smooth curves drawn here are for visualization purposes.

This paper proposes a retention time trajectory (RTT) matching method (Figure 1A) for peak identification. With peak retention times as the only input, RTT matching can identify peaks associated with any subset of target compounds, as well as filter interferents outside of said targets. An RTT (Figure 1B) is made up of a series of retention times of all compounds (peaks) in a chromatogram obtained under one set of experimental conditions (such as ambient temperature, column temperature ramping profile, carrier gas flow rate, etc.) and uniquely represents one particular condition. Similarities between the RTT for the sample under test (RTT_sample_) and those pre-installed in the library (RTT_lib_) are globally evaluated using a statistical expression, until an RTT_sample_ that best matches an RTT_lib_ is found. In contrast to most existing MS-free chromatogram-aligning algorithms, our RTT algorithm identifies peaks through simple *matching* instead of *chromatogram aligning*, that is, our method does not align the chromatogram of the sample under test against the reference chromatogram. Therefore, no mathematical warping or transformation is involved.

## 2. Retention Time Trajectory Matching Algorithm

A glossary of abbreviations and symbols is summarized in Section S1 in the Supplementary Materials.

### 2.1. Overview

Between GC analytical runs, the retention time (RT) for a given compound may drift due to perturbation of various physical factors, including ambient temperature, column temperature programing profile, and carrier gas flow rate. The influence of these perturbations on the analytes in a sample can be quite different due to their diverse characteristics (such as volatility, polarity, functional groups, etc.). Consequently, the RT drifts of the analytes in a chromatogram are often non-linear and unpredictable [21,33]. The RT deviation (ΔRT) against RT has been described using quadratic functions in PTW [20,21] or local regression fitting (LOESS) in XCMS [33], which often over-simplifies the diverse and complex nature of RT drifting. These methods are either limited to samples with the same constitutions [21] or require MS-based peak matching before final aligning [33]. In contrast, our RTT matching approach treats the RT drifts of *all* analytes (or peaks) in a chromatogram as a whole cohesive entity rather than independent individuals. Instead of fitting the RT drift with a mathematical formula, our RTT matching approach statistically compares the similarities between the RTT_sample_s and the RTT_lib_s globally.

### 2.2. Construction of a Retention Time Trajectory (RTT)

First, we describe how to construct a library containing many RTT_lib_s. Let us assume that we obtain multiple chromatograms under various experimental conditions, and each chromatogram includes *all* compounds, i.e., target compounds and internal standard compounds (if needed). The *X*-axis of Figure 1B represents the retention time (RT_X_) obtained from one of the measured chromatograms, which we call Chromatogram X. The dots of different colors along the *X*-axis represent different compounds. RTs of any pre-characterized chromatogram can be used as the *X*-axis. Similarly, the *Y*-axis represents the retention time, RT_Y_, in another chromatogram. Therefore, the set of coordinates, (RT_X,compound *i*_, RT_Y,compound *i*_), where “compound *i*” refers to a specific compound, forms a trajectory in a two-dimensional (2D) diagram (Figure 1B), which we call retention time trajectory (RTT). Each trajectory corresponds uniquely to a chromatogram obtained under a certain set of conditions (temperature ramping, flow rate, etc.), and the entire set of trajectories in the 2D diagram (i.e., the library of RTT_lib_s, Figure 1C) captures all chromatograms under various experimental conditions. One special case is that Chromatogram X itself is represented by a straight line at 45° with respect to the *X*-axis (Figure 1D), as the coordinates along the *X*- and *Y*-axis exactly match each other. Ideally, the RTT library should contain an infinite number of RTTs to reflect the infinite number of experimental conditions (for example, the flow rate may vary by only 0.0001 mL/min and ramping temperature rate may vary by 0.001 °C/min). However, as we will discuss later, a library containing a finite (small) number of RTTs is sufficient for us to correctly identify peaks in a chromatogram for the sample under test.

**Figure 2:**
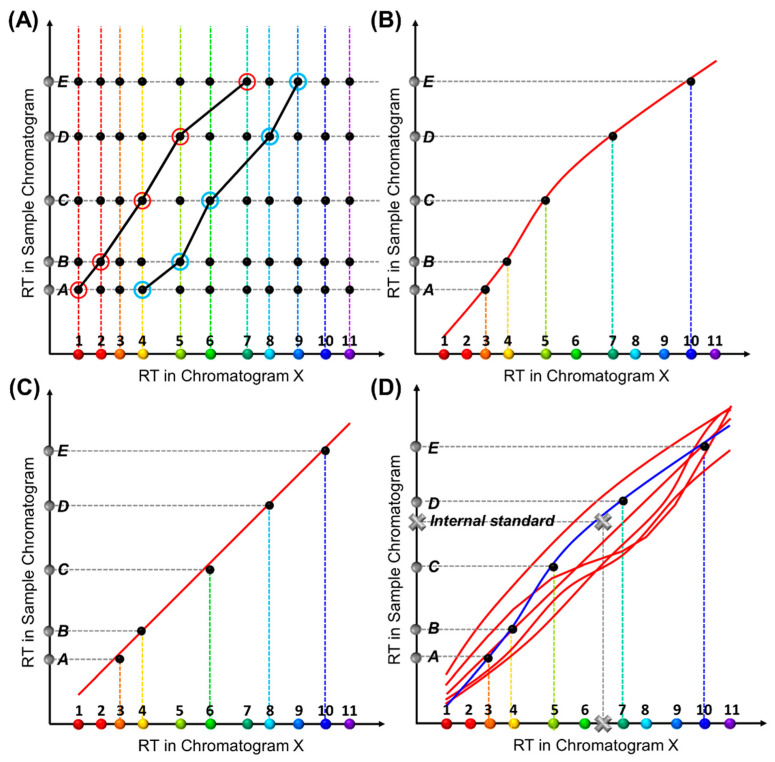
Conceptual illustration of the generation of an RTT for a sample under test, RTT_sample_. (**A**) A retention time matrix formed by RT_X_ and RT_sample_ of the same compounds. Suppose there are a total of *N_tgt_* target analytes (colored dots along the *X*-axis) and a total of *N_sample_* peaks in the sample chromatogram (grey dots denoted from A to E along the *Y*-axis). A matrix of *N_tgt_ × N_sample_* coordinates (black dots) can be formed. The two black curves show two exemplary RTT_sample_s formed by connecting one dot on each row. (**B**,**C**) Two sets of black dots fall simultaneously on two different RTT_lib_s, suggesting that two RTT_sample_s are found to match two different RTT_lib_s, which leads to two different chemical identification results. Accordingly, Peaks A-E are identified as Compounds 3, 4, 5, 7, and 10 in (**B**), and Compounds 3, 4, 6, 8, and 10 in (**C**), respectively. (**D**) An internal standard (grey cross) anchors one of the RTT_lib_s (plotted in blue), thus producing unique chemical identification.

Construction of an RTT_sample_ for a sample under test is similar (Figure 2A). Each RTT_sample_ is made up of a series of coordinates, (RT_X,compound *i*_, RT_sample,peak *j*_), where RT_sample, peak *j*_ refers to the retention time of a peak in the chromatogram obtained from the sample under test (i.e., sample chromatogram). Note that the peaks in the sample chromatogram may contain all target compounds plus some interferents or only a subset of target compounds plus some interferents. Because the chemical identities of the detected peaks are unknown before analysis, multiple RTT_sample_s may form for a given sample chromatogram, each of which corresponds to one set of peak identification results. Our goal is to eliminate all impossible RTT_sample_s and find the one that best matches one of the RTT_lib_s in the library.

### 2.3. General Description of the RTT Matching Approach

In general, our RTT matching approach involves four steps:

(1) Experimentally generate multiple chromatograms under various experimental conditions (temperature ramping profiles, flow rate, etc.) using a mixture that contains *all* target compounds and internal standards (if needed). Retention times in each chromatogram are extracted, which form the RTT_lib_s (see Figure 1C) pre-installed in the library. Note that the chemical identities of all peaks in any RTT_lib_ are known.
(2) Create all possible RTT_sample_s from the chromatogram obtained from the sample under test (see Figure 2). Note that the test sample may contain only a subset of the target compounds along with some interferents.
(3) Eliminate the RTT_sample_s that violate certain rules, which expedites computation.
(4) Compare all possible RTT_sampe_s with the RTT_lib_s in the library one by one in terms of similarities. Find the RTT_sample_ that best matches one of the RTT_lib_s and then extract the chemical identities for the detected peaks accordingly. Again, our method does not align the peaks in the sample chromatogram against those in the chromatograms in the library.

In the next section, the following two cases will be considered. (I) Only a subset of compounds of interest (target compounds) and internal standards (if needed) are present in the sample chromatogram. (II) A few chemical interferents are present in the sample chromatogram.

### 2.4. Case I: Sample Containing Only a Subset of Target Compounds

#### 2.4.1. Construction of Possible RTT_sample_s

Consider a sample under test that has no interferents present (i.e., all detected peaks are a subset of the target compounds). We assume that there are a total of *N_tgt_* target compounds and that *N_sample_* peaks are detected in the sample under test. If *N_sample_* = *N_tgt_*, then all *N_sample_* peaks can be identified easily by examining the peak elution order.

We now consider only the situation in which *N_sample_* < *N_tgt_*, that is, only a subset of analytes are present in the sample chromatogram. A matrix with *N_tgt_ × N_sample_* intersections (coordinates) is then formed in the 2D diagram (marked as black dots, i.e., coordinates in Figure 2A), because each peak in the sample under test is unknown before final identification and, in principle, can be any of the *N_tgt_* target compounds. Consequently, a total of $C\left(N_{tgt},N_{sample}\right)$ sets of RTT_sample_s can be formed by connecting one black dot in each of the *N_sample_* rows, two of which are exemplified as black lines in Figure 2A. $C\left(N_{tgt},N_{sample}\right)=\frac{N_{tgt}!}{\left(N_{tgt}-N_{sample}\right)!N_{sample}!}$ is a combinatorial number and can be extremely large when *N_tgt_* is above 20 and *N_sample_* is around half of *N_tgt_* (for example, *C*(20,10) = 184,756).

However, not all $C\left(N_{tgt},N_{sample}\right)$ RTT_sample_s are possible, and many need to be eliminated before the comparison with the RTT_lib_s in the library, which expedites computation and avoids false identifications. The elimination rules are described as follows:

(1) One target compound can only be mapped to one peak in the sample chromatogram. Therefore, any RTT_sample_ with a vertical section between any two consecutive coordinates (or intersections) in the 2D diagram should be eliminated, as illustrated in Figure S2A.
(2) The elution order should be preserved. Therefore, any RTT_sample_ with a section that has a negative slope between two consecutive coordinates (i.e., opposite elution order in library and sample chromatograms) should be eliminated. This is illustrated in Figure S2B.
(3) The RT drifts arise from minor perturbation, and the resulting deviations (ΔRT) should be small values within a certain range. Therefore, only coordinates falling within the cutoff range of RT_sample_ ± Δt should be considered (Figure S2C). The value of Δt can be estimated empirically. For example, sample chromatograms with larger drifts require sufficiently large Δt (e.g., larger than typical RT drifting range in the RTT library). Note that the RTT matching algorithm is still able to effectively identify the peaks even without applying this criterion, but a reasonable estimation of Δt significantly reduces the computational cost by narrowing down possible RTT_sample_s.

Once all possible RTT_sample_s are formed (and all impossible RTT_sample_s are eliminated), each individual RTT_sample_ should be compared with all RTT_lib_s in the library to find the best-matched RTT_sample_. In other words, we need to find which set of coordinates in Figure 2A falls on one red RTT_lib_ in Figure 1C. Assuming a total of *n_lib_* RTT_lib_s stored in the library and *n_sample_* possible RTT_sample_s generated from the sample chromatogram, *n_lib_* × *n_sample_* pairs of RTT_lib_ and RTT_sample_ are formed and compared with each other. For example, by comparing the trajectories in Figure 1C, two groups of black dots, which are composed of two different RTT_sample_s, fall simultaneously on two different RTT_lib_s (Figure 2B,C) and yield different chemical identification results for Peaks C and D. These are either Compounds 5 and 7 (Figure 2B) or 6 and 8 (Figure 2C).

To circumvent this, we introduce internal standard compounds (i.e., internal standards) outside of the list of target compounds to anchor the RTT_lib_s. In both RTT_lib_ library preparation and actual measurement of the test sample, the internal standard(s) are spiked into the mixture containing *all* target compounds (for RTT_lib_ library preparation) or the sample under test. The peaks corresponding to these standards are identified during the data preprocessing and then used to generate the RTT along with all other peaks. As depicted in Figure 2D, when an internal standard (marked as a grey cross) is introduced, only one of the two RTT_lib_s can be anchored (blue line) and thus a unique set of identification results is obtained.

Note that while our RTT matching method, as shown later, works well even without using any internal standard, introduction of an internal standard(s) can further increase identification accuracy and significantly expedite computation by narrowing down possible RTT_sample_s and RTT_lib_s due to the following reasons. (1) All possible RTT_sample_s and all RTT_lib_s must go through the coordinate(s) formed by the internal standard(s). (2) Because the elution order is preserved, the whole 2D diagram can be divided into small regions determined by the internal standards’ coordinates, and only the RTT_sample_s falling within these regions are possible candidates (Figure S3A). (3) When there is only a single analyte in the test sample, identification of this peak in the chromatogram is nearly impossible without internal standard(s), as formation of an RTT requires at least two coordinates. The addition of one or more coordinates resulting from the internal standards allows for the creation of the RTT_sample_s with more accurate identification of the single peak. (4) Misidentification can be significantly reduced even when the chromatogram-to-chromatogram RT drift of the same compound is greater than the distance between two adjacent peaks (Figure S3B), which has long been the bottleneck of many peak-matching or profile-aligning algorithms [33]. In practice, internal standards can be strategically positioned in the region with more drastic variations to more effectively narrow down RTT_sample_ and RTT_lib_ selection (Figure S3C). Note that, similar to all internal-standard-based chromatographic analysis methods, the addition of internal standards might potentially worsen the co-elution issues with the neighboring target compounds. To avoid this, internal standards whose RTs fall in the chromatogram sections with low peak densities are preferred.

Internal standards have commonly been utilized by many aligning algorithms [34,35], in which RT drift is corrected by firstly dividing the chromatogram into multiple sections delineated by the standards and secondly applying linear stretching/compressing in each section (Figure S4). However, these methods cannot account for the various non-linear drifts that often occur between any two standards. To make the linear stretching/compressing more accurate, more internal standards need to be introduced to reduce the section size to the point that linear approximation within each section is valid. This makes both sample preparation and peak identification much more complicated. More advanced techniques employ polynomial fitting within each section to account for non-linearity. However, polynomial fitting highly depends on experimental conditions and is required for each section in a chromatogram, thus hampering automation in peak identification. In contrast, as discussed in detail later, our approach compares the retention times of the chromatogram (or its corresponding RTT) globally, which automatically takes into account any non-linearities within each section. To demonstrate the advantages of RTT matching, we use the same sample and internal standards to compare the performance of the RTT matching approach and linear warping (see examples in Table S5), as well as correlation optimized warping (COW, see examples in Figures S8 and S9, and Table S4 in the Supplementary Materials).

#### 2.4.2. Statistical Method for RTT Similarity Comparison—Least Mean Squared Residual (MSR)

**Figure 3:**
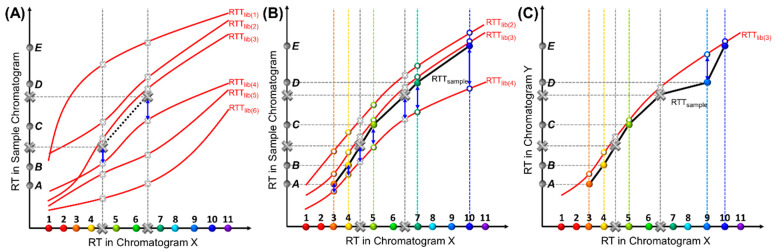
MSR or SSR calculation. (**A**) Illustration of calculating the SSRst^d^ between an RTT_sample_ and RTT_lib(4)_ using the internal standards for RTT_libs_ screening. All RTT_samples_ must pass through the two large solid grey crosses, which indicate the RTs of the internal standards in the sample chromatogram. The smaller hollow grey crosses indicate the RTs of the internal standards in the RTT_libs_. The internal standard retention time residuals are marked by blue arrows. Half of the RTT_libs_ with the lowest SSRst^d^s (i.e., RTT_lib(2)_, RTT_lib(3)_, and RTT_lib(4)_) are kept and used for the next step described in (**B**). (**B**) Calculation of the MSR between an RTT_sample_ and an RTT_lib_ screened in (A) using both target compounds and internal standards. This process quantifies the similarities between the two RTTs under comparison (i.e., one RTT_sample_ and one RTT_lib_). The RTT_sample_ (black curve) is formed by pairing Peaks A to E with target Compounds 3, 4, 5, 7, and 10. The retention time residual of the same compound is marked by a blue arrow. (**C**) Interferent identification. One RTT_sample_ (black curve) is formed by pairing Peaks A to E with target Compounds 3, 4, 5, 9, and 10, respectively, and is further compared with RTT_lib(3)_ (red curve). The retention time residual between Peak D and Compound 9 is much larger than those of other pairs. The physical interpretation is that the coordinates associated with Peaks A, B, C, and E in one RTT_sample_ closely match those on RTT_lib(3)_. However, Peak D deviates far from RTT_lib(3)_, likely identifying Peak D as an interferent, and identifying Peaks A, B, C, and E as Compounds 3, 4, 5, and 10, respectively.

As mentioned previously, while coordinates in the *X*-axis are discrete, their variation along the *Y*-axis is continuous due to continuously varying experimental conditions. In theory, the number of the RTT_lib_s is infinite. In practice, only a limited number of conditions, and, hence, a limited number of RTT_lib_s, can be characterized and stored. Consequently, while the RTT_sample_ for the sample under test may not exactly match any RTT_lib_ stored in the library, the most similar one can still be easily found. To globally compare similarities between an RTT_sample_ and an RTT_lib_, we calculate the mean squared residuals (*MSR*) of RTs from the same compounds between these two trajectories, as illustrated in Figure 3. A smaller *MSR* indicates higher similarity between two trajectories, as exemplified in Figure 3B, where RTT_lib(3)_ is the most similar to the RTT_sample_ in the figure. The *MSR* is normalized (or scaled) from the sum of squared residuals (*SSR*) by the total number of paired compounds in order to ensure that it does not grow as the number of pairs grows. This is important when we need to compare RTT_sample_s with different numbers of target compounds; for example, when an RTT_sample_ of six target compounds is compared with an RTT_sample_ of five target compounds plus one interferent (the case of interferent identification will be discussed later).

In order to expedite the computation, it is not necessary to compare each RTT_sample_ with all RTT_lib_s. Instead, we can first use internal standard RTs to anchor the best matching sets of RTT_lib_s by calculating the *SSR* of the internal standards (*SSRst^d^*), as shown in Figure 3A. Because all possible RTT_sample_s must go through the coordinates formed by the internal standards, they have the same *SSRst^d^* for a given RTT_lib(*i*)_, where *i* refers to a specific pre-characterized RTT_lib_. Assuming that there are a total of *N_std_* internal standards, the *SSRst^d^* between any RTT_sample_ and an RTT_lib(*i*)_, denoted as $SSR_{lib\left(i\right)}^{std}$, can be calculated as

$$SSR_{lib\left(i\right)}^{std}=\overset{N_{std}}{\underset{k=1}{\sum }}{\left(RT_{lib\left(i\right)}^{std\left(k\right)}-RT_{sample}^{std\left(k\right)}\right)}^{2},$$

where $RT_{lib\left(i\right)}^{std\left(k\right)}$ is the retention time of one internal standard, *k*, in RTT_lib(*i*)_, and $RT_{sample}^{std\left(k\right)}$ is the retention time of the same internal standard in the sample chromatogram. The *SSRst^d^* of all RTT_lib_s are sorted in ascending order, with the top ones, which have the least *SSRst^d^*s, giving the potentially matched RTT_lib_s. All other RTT_lib_s in the library, which have higher *SSRst^d^*s, can be eliminated.

Next, the RTT_sample_ and RTT_lib_ are further compared, based on retention times of both internal standards and target compounds, by sorting the *MSR* (Figure 3B). In this step, only the top RTT_lib_s (e.g., the first half or the top 20 RTT_lib_s) with the lowest *SSRst^d^*s are selected. Assuming that, in addition to *N_std_* internal standards, there are *N_sample_* peaks to be identified in the test sample, the *MSR* between one RTT_lib_ (denoted as RTT_lib(*i*)_) and one RTT_sample_ (denoted as RTT_sample(*j*)_) can be calculated as

$$\begin{array}{c} MSR=\frac{{\mathrm{SSR}}_{lib\left(i\right),sample\left(j\right)}}{N_{std}+N_{sample}}\\ \;\;\;\;\;\;\;\;\;=(SSR_{lib\left(i\right)}^{std}+\overset{N_{sample}}{\underset{l=1}{\sum }}{\left(RT_{lib\left(i\right)}^{compound\left(l\right)}-RT_{sample\left(j\right)}^{compound\left(l\right)}\right)}^{2})/(N_{std}+N_{sample)}{,}_{\;} \end{array}$$

where $RT_{lib\left(i\right)}^{compound\left(l\right)}$ is the retention time of the Compound *l* in RTT_lib(*i*)_, and $RT_{sample\left(j\right)}^{compound\left(l\right)}$ is the retention time of a peak in the sample chromatogram that is hypothetically assigned to the same compound (i.e., Compound *l*) in RTT_sample(*j*)_. Note that all RTT_sample_s have the same retention time for each peak, but each peak can hypothetically be paired with a different compound in different RTT_sample_s, which has been discussed previously (e.g., Figure 2A). The *MSR* of all RTT_lib_s are sorted in ascending order. The first in the list has the minimum *MSR* value denoted as *MSR_sample(j), min_*, which is generated by the RTT_lib_ that best matches RTT_sample(*j*)_.

Because each RTT_sample_ is formed by pairing the detected peaks with one set of target compounds, it represents one set of peak identification results. For any RTT_sample_, which we call RTT_sample(*j*)_, the best-matched RTT_lib_ can be found by screening all RTT_lib_s in the library and finding the one that generates *MSR _sample(j)_*_, min_. If there is another RTT_sample_, denoted as RTT_sample(*k*)_, that has *MSR_sample(k),min_* smaller than *MSR_sample(j),min_*, it means that RTT_sample(*k*)_ is a better match with one of the RTT_lib_s in the library. Therefore, the corresponding peak identification results from RTT_sample(*k*)_ are more accurate than those from RTT_sample(*j*)_.

### 2.5. Case II: Sample Containing Interferents

In targeted analysis, interferents are the compounds not on the list of the target compounds and need to be filtered out (or reported). In our algorithm, two criteria are used to identify the presence of interferents. First, for a particular peak in the sample chromatogram, if none of the retention time values in the RTT_lib_s fall in the range of RT_peak_ ± Δt, this peak is identified as an interferent. The value of Δt can be chosen empirically and can be set higher than a typical retention time drift range. Second, for one particular pair of RTT_lib(*i*)_ and RTT_sample(*j)*_, and if the squared residual between one peak (for example, Peak *D* in Figure 3C) in RTT_sample(*j*)_ and its paired compound (Compound 9 in Figure 3C) in RTT_lib(*i*)_ is much larger (e.g., twice) than *MSR_lib(i),sample(j)_*, it is highly likely that this peak is an interferent. The validity of this approach lies in the fact that all other coordinates formed by the detected peaks and their paired target compounds match well with RTT_lib(*j*)_, except for the one formed by Peak *D* paired with Compound 9. A new *MSR_lib(i),sample(j)_* is then calculated by normalizing *SSR_lib(i),sample(j)_*, which excludes residuals from all identified interferents, with $N_{std}+N_{sample}-N_{interf}$ ($N_{interf}$ is the total number of identified interferents). Based on this, all possible peak identification results, with or without interferents, can be ranked by *MSR*s. The results with the smallest *MSR* give the highest confidence level.

## 3. Experimental Section

### 3.1. Chromatogram Generation

**Figure 4:**
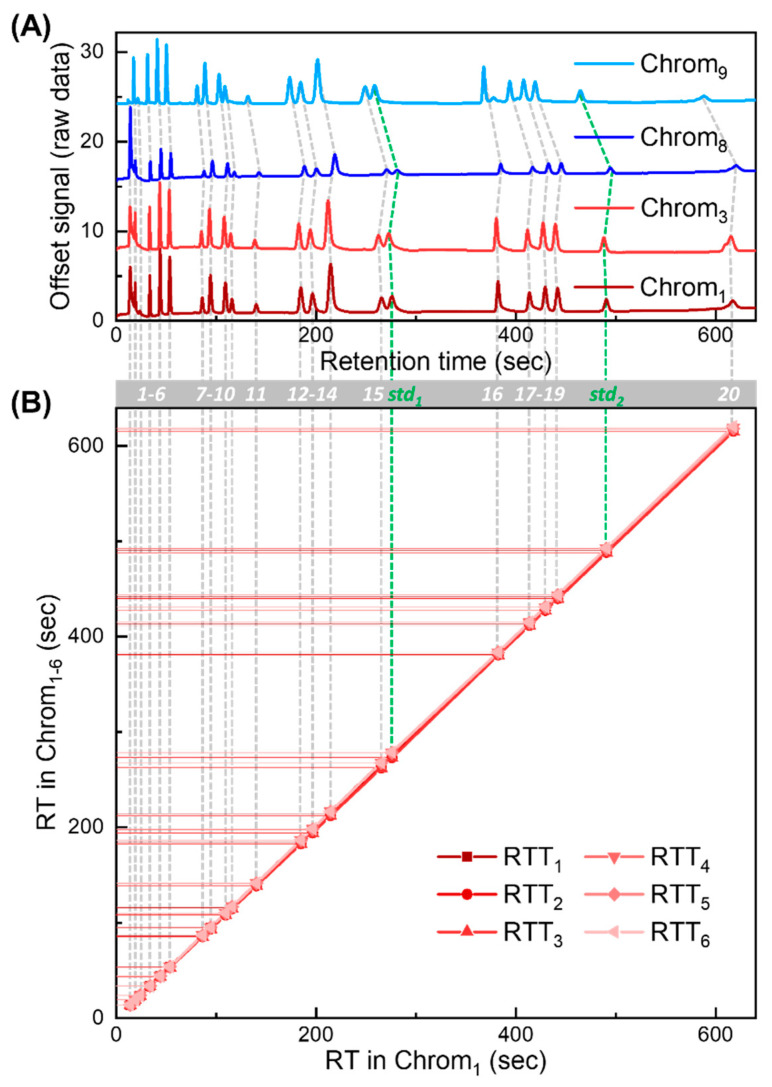
Experimentally generated chromatograms and corresponding RTT_lib_s for algorithm validation. (**A**) Four exemplary chromatograms (Chrom_1_, Chrom_3_, Chrom_8_, and Chrom_9_) obtained experimentally. There are a total of 22 detected peaks in each chromatogram. A total of 2 out of the 22 peaks are treated as internal standards (labeled as std_1_ and std_2_ in green). The remaining peaks are treated as target compounds for peak identification in targeted analysis. Peaks of the same compounds are labeled with compound IDs from 1 to 20 (grey bar). (**B**) RTT_lib_ library formed by RTT_lib_s (RTT_1–6_) generated from Chrom_1–6_. The *X*-axis represents retention time in Chrom_1_. The *Y*-axis represents retention times in Chrom_1–6_. The retention time deviation (ΔRT) of Chrom_1–9_ against the RT in Chrom_1_ is plotted in Figure S5.

Our RTT approach is validated using nine chromatograms obtained with NovaTest P300 GC provided by Nanova Environmental, Inc., which is equipped with a 6 m long Rtx-VMS column (Restek, Bellefonte, PA, USA) and a microfluidic photoionization detector developed in-house [36]. The chromatograms were generated under the same nominal experimental setting (carrier gas: helium; flow rate: 3.5 mL/min; temperature programming profile: 40 °C held for 5 min, ramped to 70 °C at 30 °C/min, held for 2 min, then ramped to 150 °C at 30 °C/min, and held for 1 min). The injected mixture is part of EPA Method TO-14. Exemplary chromatograms are presented in Figure 4A, and corresponding peak information is listed in Table S1.

### 3.2. Chromatogram Preprocessing

Detection of a peak in a chromatogram is accomplished by scanning for local maxima and the associated peak apex positions (i.e., retention times) [37]. A series of retention times are extracted, which are used to form the RTT_lib_s or RTT_sample_s in the next section. Therefore, the cumbersome chromatographic data (a large 2D array of detection signals) are converted to a simple list of retention times, which significantly reduces data storage and processing workload. Extensive preprocessing (e.g., baseline removal) and broad background variations can also be eliminated because only the local maxima (i.e., peak apexes) are extracted.

## 4. Validation Results

Out of nine experimentally generated chromatograms, six chromatograms (denoted as Chrom_1–6_) are used in the library, forming RTT_lib_s (Figure 4B). The remaining three (denoted as Chrom_7–9_) are used to generate tests to validate our approach in various scenarios.

Detailed validation test design is described in Section S2 in the Supplementary Materials. There are a total of 22 peaks in each measured chromatogram, among which 20 are treated as target compounds and the other two are used as the internal standards. The retention times and compound IDs of Chrom_1_ are summarized in Table S1. The RT deviation (ΔRT) against the RT in Chrom_1_ for all chromatograms (Chrom_1–9_) is plotted in Figure S5, showing strong non-linear drifting behavior. Note that while the chemical names for most compounds are provided by the vendor and further confirmed by injecting individual compounds, which are given in the third column in Table S1, the chemical identities of the first and the third eluted peaks are unknown (which might result from contamination and are only designated as ID 1 and ID 3, respectively). Nevertheless, the results presented in this work remain the same regardless of whether the chemical names of those compounds are known.

In total, $3\times \sum_{i=1}^{20}\left[C\left(20,i\right)\right]=3.15\times 10^{5}$ validation tests were generated from Chrom_7–9_, covering *all* subsets of the 20 target compounds (ranging from single compounds to 20 compounds). Moreover, three additional validation tests were generated, representing samples with a subset of target compounds *and* interferent(s). In all $3.15\times 10^{5}$ validation tests, peak identifications achieved 100% accuracy. Here, we present detailed results of 11 representative tests, covering various MS-free chromatographic analysis scenarios, i.e., (1) different levels of retention time (RT) drift, (2) different numbers of the target compounds in the sample under test, and (3) samples containing interferents.

### 4.1. Identification of Target Compounds in Sample under Test without Interferents

To validate our algorithm, we first discuss the scenarios in which no interferents are present and all the detected peaks are a subset of the target compounds. As shown in Table 1, based on the RTs in Chrom_7_ and Chrom_8_, six groups of RTs are generated, three from Chrom_7_ (Table 1A) and three from Chrom_8_ (Table 1B), for six tests. They represent six different samples under test that contain various subsets of the target compounds (the number of the target compounds ranges from 1 to 13 out of a total of 20 target compounds), along with two internal standards (std_1_ and std_2_). Note that the retention time deviation in Chrom_7_ is within the range of the RT deviations in the chromatograms (Chrom_1–6_) stored in the library, whereas the retention time deviation in Chrom_8_ is slightly out of this range (Figure S5). For each test, the four best peak identification results are enumerated based on the *MSR* ranking (Table 1). The peaks in all six tests are successfully identified with the top result (i.e., smallest *MSR*) producing 100% accuracy. The 2nd-4th best results in each test also correctly identify most of the peaks with the best ones giving 100% accuracy and the worst ones misidentifying only one peak. Note that even a single-species sample (Compound 7 in Test 6) can be correctly identified due to the use of internal standards, despite it being very close to neighboring Compound 8. This is impossible for all warping-based chromatogram-aligning approaches, because the peak has nothing to be aligned with.

### 4.2. Identification of Target Compounds in Sample under Test with Interferents

Another three validation experiments (Tests 7–9 in Table S2) were generated based on the RTs in Chrom_8_, which mimic scenarios in which both a subset of target analytes and interferents are present in the sample under test. In Tests 7 and 8, one hypothetical interferent peak was added at 340 s (for Test 7) and 449 s (for Test 8). In particular, the interferent peak at 449 s was very close to the target Compound 19. In both cases, the top identification result successfully identified all target compounds and singled out interferent related peaks with 100% accuracy. In Test 9, two hypothetical interferent peaks were added at 62 s and 395 s, which were very close to target Compounds 6 and 16, respectively. Both interferents and all target compounds were correctly identified. Like all other RT-based peak identification methods discussed previously, the RTT-matching-based interferent identification works only when RT_interferent_ is sufficiently different from those of target compounds. If RT_interferent_ is the same as or extremely close to any target compound, the interferent cannot be identified. Additionally, the majority of the peaks in the sample under test should be the target compounds. If most peaks are interferents and only a few target compounds are present, the validity of our method may decrease, because the residuals of most peaks are very large. One way to circumvent these issues is to further enrich the RTT library, either experimentally or through the RTT hybridization discussed in Section 4.3. Introducing more internal standards may also be helpful.

It is also worth noting that the RTT matching approach is intended for the scenarios where RT drifts are caused by only minor fluctuations in experimental conditions, and, therefore, the elution order is expected to be preserved among the measurements. When the experimental condition varies drastically (e.g., major changes in the device settings or ambient temperatures), the elution order may switch. Therefore, a new RTT library needs to be constructed under the new experimental conditions to avoid misalignment/misidentification in the RTT matching approach.

The above two issues (i.e., serious co-elution and elution order change) have always been the bottleneck for all existing MS-free chromatogram-aligning algorithms. MS (and other spectroscopic methods such as infrared absorption spectroscopy and Raman spectroscopy) would potentially be needed for peak identification in these cases.

### 4.3. Identification of Target Compounds in Sample under Test with Severe RT Drifts

An ideal RTT library should contain all possible RTT_lib_s that cover all possible drift-inducing conditions. If the library has only a limited number of RTT_lib_s and when the RT drift in a sample chromatogram (or RT deviation) exceeds the RT deviations covered by the RTT_lib_s, peak misidentification may occur, as exemplified by Tests 10 and 11 in Table S3. One method to expand the library is to experimentally generate as many RTT_lib_s as possible by varying the experimental conditions around the nominal conditions. However, this is extremely labor-intensive and difficult to realize. Alternatively, new RTT_lib_s can be generated and added to the library by linearly hybridizing existing experimentally obtained RTT_lib_s. This method is valid because the retention time drift is caused by minor fluctuations of system’s physical factors, and such a small perturbation in the retention time of one particular compound from one state (one RT) to another state (another RT) can be simplified as linear variation. RTT linear hybridization can be performed using two, three, or more existing experimentally obtained RTT_lib_s, i.e.,

$$C_{1}\times RT_{lib\left(a\right)}^{compound\left(i\right)}+C_{2}\times RT_{lib\left(b\right)}^{compound\left(i\right)},\;$$

or

$$C_{1}\times RT_{lib\left(a\right)}^{compound\left(i\right)}+C_{2}\times RT_{lib\left(b\right)}^{compound\left(i\right)}+C_{3}\times RT_{lib\left(c\right)}^{compound\left(i\right)},$$

where *C*_1_, *C*_2_, and *C*_3_ are the linear coefficients, and $RT_{lib\left(a\right)}^{compound\left(i\right)}$ refers to the retention time for Compound *i* in one of the RTT_lib_s (i.e., RTT_lib(a)_). The hybridization can easily generate more RTT_lib_s of the intermediate states that may be difficult to obtain experimentally (due to either time limitations and/or difficulty in realizing the exact experimental conditions), which significantly increases the tolerance to more severe RT drifts. Note that the RT variation of one particular compound from one state to another is simplified to be linear, although ΔRT against RT in one chromatogram is generally non-linear.

In the current work, in order to validate the hybridization method, we use two-RTT hybridization based on the following three formulas: (1) $\left(RT_{lib\left(a\right)}^{compound\left(i\right)}+RT_{lib\left(b\right)}^{compound\left(i\right)}\right)/2$, (2) $2RT_{lib\left(a\right)}^{compound\left(i\right)}-RT_{lib\left(b\right)}^{compound\left(i\right)}$, and (3) $2RT_{lib\left(b\right)}^{compound\left(i\right)}-RT_{lib\left(a\right)}^{compound\left(i\right)}$. These are used to enrich the RTT_lib_s in the library (see Figure S6), where RTT_lib(a)_ and RTT_lib(b)_ are selected from the Chrom_1–6_ that are experimentally generated. Two tests (Tests 10 and 11) are generated based on Chrom_9_, which has much more severe RT drift than Chrom_7–8_ and any pre-characterized chromatograms (Chrom_1–6_) in the library (see Figure S5). As shown in Table S3, when the RTT library contained only the experimentally generated RTT_lib_s, it failed to correctly identify all peaks in either Test 9 or 10. In contrast, with more RTT_lib_s added through hybridization, all peaks were successfully identified with 100% accuracy. The accuracy of the 2nd–4th identification results in both tests were also increased.

### 4.4. Comparison with Other Chromatogram-Aligning Approaches

In order to compare peak identification performance with other chromatogram-aligning approaches, parts of the above validation tests are also performed with the whole-chromatogram-based COW [19,20] method and two peak-list-based aligning methods, namely the internal-standard-based linear warping approach and fast PTW [22]. Again, it is worth emphasizing that our RTT algorithm identifies peaks through matching instead of chromatogram aligning.

For a chromatogram-aligning method to work, we use Chrom_1_ (or its corresponding peak list) as the reference for other chromatograms (i.e., sample chromatograms) to align with. Tests 5 and 7 (based on Chrom_8_), which represent samples without and with an interferent, respectively, are used to evaluate COW and linear warping. The retention times after COW or linear warping [35] are summarized in Table S4 and Table S5, respectively. Note that in the reference chromatogram (i.e., Chrom_1_), all the peaks are present, including *all* target compounds and internal standards. The sample chromatograms, which are to be aligned and identified, are reconstructed from Chrom_8_, but contain only the peaks listed in Tests 5 and 7. The remaining peaks in Chrom_8_ are replaced with the baseline (see Section S3 in the Supplementary Materials for details of sample chromatogram reconstruction).

The COW aligning results can be highly parameter-dependent [19]. When the peak compositions in the reference and sample chromatograms are the same, optimal parameters can be easily chosen so that the apex positions of the peaks of the same elution order in the sample chromatogram and the reference chromatogram coincide. However, in the presence of only a subset of the target compounds in the sample under test (as in Tests 5 and 7), the peak in the sample chromatogram has no specific target peak to align to, and, therefore, the COW alignment parameter selection becomes dubious. Similarly, the interferent peak (as in Test 7) might be misaligned to one of the target compound peaks in the reference chromatogram. Multiple COW alignments with various tuning parameters (slack and correlation power) were conducted. The identification results based on the RTs extracted from the aligned Chrom_sample_ are summarized in Table S4. Out of the seven peaks in Test 5, the best COW aligning correctly identifies only five peaks (slack = 4 and correlation power = 3) and the worst aligning fails to align any of the peaks (slack = 1 and correlation power = 1; slack = 2 and correlation power = 2). For Test 7, the highest identification accuracy reaches only 50% (slack = 4 and correlation power = 3) and the worst aligning fails with the whole chromatogram (slack = 1 and correlation power = 1; slack = 2 and correlation power = 2).

For the internal-standard-based linear warping method (Table S5), the same internal standards (std_1_ and std_2_) were employed. After alignment, none of the target compound-associated peaks yields the same RT as in the reference chromatogram (Chrom_1_). Therefore, peak identification for target compounds fails when RT values are compared (all peaks are identified as interferents, except the interferent peak itself).

Additionally, we compared the peak identification performance of the fast PTW algorithm [22], which is a further development of the original PTW [21], and the algorithm input was the chromatogram peak list (retention times and peak heights). The peak lists in Chrom_1_, which was treated as the reference chromatogram, and Chrom_8,9_, which were used to generate Tests 5, 7, 10, and 11, are summarized in Table S6A. The time warping results (order = 2 or 3) using the full peak lists in Chrom_8,9_ are summarized in Table S6A. Although misalignments were greatly reduced after fast PTW aligning, sub-second to seconds of RT difference for the same compound persists. Table S6B summarizes the fast PTW aligning results (order = 2 or 3) of Tests 5, 7, 10, and 11. Tests 5, 10, and 11 represent samples with only a subset of target compounds; Test 7 represents a sample with a subset of target compounds and an interferent. The fast PTW fails to identify the correct peaks in all four tests. In comparison with the reference chromatogram, the RT differences in the same compound in the warped sample peak list fell in the range of sub-seconds (which could be solved by data clustering) to hundreds of seconds (which completely fails in peak identification). We also noticed that the warped RTs of some peaks gave unreasonable negative values (e.g., Compound 1 in Test 10, with order = 2, and Compound 17 in Test 10, with order = 3).

### 4.5. Further Validation with Fruit Metabolomics Data

Two publicly available liquid chromatography (LC) fruit metabolomics datasets from the Metabolights repository (http://www.ebi.ac.uk/metabolights, accessed on 10 March 2021) with identifiers MTBLS99 and MTBLS85 are used to generate additional validation tests. This demonstrates the application of the RTT matching algorithm to complicated samples and other chromatographic techniques. The first dataset consists of LC measurements of a pooled sample that was injected regularly as a quality control during the measurements of apple extracts (Figure S10A). The second dataset is from LC measurements of carotenoids in grape samples (Figure S10B). The test design and results are summarized in Section S4 in the Supplementary Materials. In all of the two trillion designed validation tests, all peaks associated with the target compounds were correctly identified.

## 5. Discussion and Conclusions

We have described in detail and validated a new RTT matching approach for the identification of target compounds and the detection of interferents. This approach has the following advantages. First and foremost, the matching is conducted between the entire trajectories (RTT_sample_ and RTT_lib_) rather than between the individual peaks in the sample and those in the reference chromatogram. Second, simple statistics are used, which avoids time-consuming training or feature extraction used in machine learning [28,31,32]. The *MSR* is adopted to describe the similarities between RTTs, which works well with the chromatograms obtained in this article. Other statistical approaches, such as linear regression, can also be introduced within the framework of RTT matching. Third, hybridization of RTT_lib_s greatly expands the RTT library, which not only increases the tolerance to more serious drifting but also significantly reduces the cost for RTT_lib_ generation through actual experimentation. In this work, each hybridized RTT_lib_ is generated out of two experimentally obtained RTT_lib_s. Depending on the complexities of the subjects to be analyzed, a larger number of experimentally obtained RTT_lib_s could be used, and the linear coefficients could be further tuned. Fourth, the only input variables are the retention times of each peak instead of the whole peak profile or mass spectra, making the RTT approach insensitive to concentration or background variations and eliminating the need for bulky instruments, such as mass spectroscopy. All the above features make RTT matching highly amenable to automation with a low computation cost. Additionally, the method described here can be easily translated to other chromatographic techniques (e.g., ion exchange chromatography) and has great potential to be applied to other spectral data (e.g., nuclear magnetic resonance spectroscopy). Finally, the introduction of internal standards, though contributing to increasing identification accuracy and computation efficiency, are not always necessary. When an increased number of target compounds are present in the sample under test, the contribution of the standard(s) becomes less prominent. To validate this, 10 of the validation test examples in the above discussions, except the single-species sample in Test 6, have been re-performed with RTT matching without the use of any internal standards. All of the peaks were correctly identified. Our current work is focused on the RTT matching for one-dimensional chromatography. However, the same idea and approach can be extended to multi-dimensional chromatography, such as comprehensive 2D GC.

Finally, it is worth noting that the applications of the RTT matching approach are not limited to just peak identification. The RTT matching approach can help in chromatogram aligning. Briefly, one can choose any RTT_lib_ as the reference chromatogram to extract RTs of the target compounds. Each peak in the sample chromatogram is identified via the RTT matching approach. The peak profile can be fitted by the exponentially modified Gaussian (EMG) model, with apexes shifted to the positions as in the reference. Aligned sample chromatograms can be formed by the summation of individual EMG reconstructed peaks. A detailed description and an illustration are discussed in Section S5 and Figure S11.

## Figures and Tables

**Table 1:** Algorithm experimental design and peak identification results with a sample containing only the target compounds. The peaks listed in Tests 1–3 are generated from Chrom_7_; the peaks listed in Tests 4–6 are generated from Chrom_8_. An asterisk “*” denotes peak misidentification.

**(A)**																	
**Test data generated from Chrom_7_**	**Test 1**	**Retention time (s)**			13.8	19	33.5	43.7	85.6	108.5	115	183.6	213.3		412.7		616
		**Compound ID**			1	2	4	5	7	9	10	12	14		17		20
		**Ranking**	**MSR**	**Accuracy**	**Individual peak identification result**												
		1st	0.57	100%	1	2	4	5	7	9	10	12	14		17		20
		2nd	0.71	100%	1	2	4	5	7	9	10	12	14		17		20
		3rd	0.91	100%	1	2	4	5	7	9	10	12	14		17		20
		4th	2.2	90.9%	1	3 *	4	5	7	9	10	12	14		17		20
	**Test 2**	**Retention time (s)**			19	33.5	43.7	85.6	93.8	108.5	115	139.3	195.2	213.3	264	381.3	412.7
		**Compound ID**			2	4	5	7	8	9	10	11	13	14	15	16	17
		**Ranking**	**MSR**	**Accuracy**	**Individual peak identification result**												
		1st	0.67	100%	2	4	5	7	8	9	10	11	13	14	15	16	17
		2nd	0.74	100%	2	4	5	7	8	9	10	11	13	14	15	16	17
		3rd	1.14	100%	2	4	5	7	8	9	10	11	13	14	15	16	17
		4th	2.15	92.3%	3 *	4	5	7	8	9	10	11	13	14	15	16	17
	**Test 3**	**Retention time (s)**			85.6		108.5		195.2			381.3			428.2		
		**Compound ID**			7		9		13			16			18		
		**Ranking**	**MSR**	**Accuracy**	**Individual peak identification result**												
		1st	0.88	100%	7		9		13			16			18		
		2nd	0.94	100%	7		9		13			16			18		
		3rd	1.48	100%	7		9		13			16			18		
		4th	5.74	100%	7		9		13			16			18		
**(B)**																	
**Test data generated from Chrom_8_**	**Test 4**	**Retention time (s)**			34.1	44.6	96.2	111.4	142.9	188.4		218.6		384.8		432.6	
		**Compound ID**			4	5	8	9	11	12		14		16		18	
		**Ranking**	**MSR**	**Accuracy**	**Individual peak identification result**												
		1st	2.42	100%	4	5	8	9	11	12		14		16		18	
		2nd	2.86	100%	4	5	8	9	11	12		14		16		18	
		3rd	3.34	100%	4	5	8	9	11	12		14		16		18	
		4th	5.06	88.9%	4	5	8	10 *	11	12		14		16		18	
	**Test 5**	**Retention Time (s)**			87.9		111.4		218.6			384.8			432.6		
		**Compound ID**			7		9		14			16			18		
		**Ranking**	**MSR**	**Accuracy**	**Individual peak identification result**												
		1st	2.99	100%	7		9		14			16			18		
		2nd	3.74	100%	7		9		14			16			18		
		3rd	4.28	100%	7		9		14			16			18		
		4th	7.14	80%	7		10 *		14			16			18		
	**Test 6**	**Retention time (s)**			87.9												
		**Compound ID**			7												
		**Ranking**	**MSR**	**Accuracy**	**Individual peak identification result**												
		1st	3.80	100%	7												
		2nd	5.10	100%	7												
		3rd	5.80	100%	7												
		4th	16.75	100%	7												

## Data Availability

Not applicable.

## Supplementary Materials

The following supporting information can be downloaded at https://www.mdpi.com/article/10.3390/s23136029/s1 [19,20,22,37,38,39,40], Figure S1: Conceptual illustration of the binning approach. Figure S2: Rules to eliminate impossible RTT_sample_s. Figure S3: Use of internal standards. Figure S4: Conceptual illustration of peak aligning with the internal-standard-based linear stretching/compressing approach using the RTT 2D diagram. Figure S5: Retention time deviation (ΔRT) of Chrom_1–9_ against the RT in Chrom_1_. Figure S6: Demonstration of RTT_lib_s hybridization. Figure S7: Illustration of algorithm validation test design using Chrom_8_. Figure S8: Peak identification using COW aligning with a sample containing a subset of target compounds (Test 5). Figure S9: Peak identification using COW aligning with a sample containing a subset of target compounds plus one interferent (Test 7). Figure S10: Fruit metabolomics chromatograms for RTT peak identification verification. Figure S11: Chromatogram aligning enabled by RTT matching. Table S1: Peak retention times, assigned compound IDs, and compound names in Chrom_1_. Table S2: Algorithm validation tests and the corresponding peak identification results. Table S3: Algorithm validation tests and the corresponding peak identification results when a sample chromatogram has severe RT drift issues. Table S4: Peak identification performance comparison with COW. Table S5: Peak identification performance comparison with internal-standard-based linear warping. Table S6: Peak identification performance comparison with fast PTW.
