# Supplementary material for: Retention Time Trajectory Matching for Peak Identification in Chromatographic Analysis
Source: Sensors (Basel). 2023 Jun 29;23(13):6029. doi: 10.3390/s23136029 (PMC10347159; doi:10.3390/s23136029)
Supplement: Supplementary file 1 [file sensors-23-06029-s001.zip › sensors-2434341-supplementary.pdf]

Supplementary Information to

Retention time trajectory matching for peak identification in chromatographic analysis

Wenzhe Zang [1,2,3], Ruchi Sharma [1,2,3], Maxwell Wei-Hao Li [1,2,3,4], and Xudong Fan [1,2,3,*]

[1]Department of Biomedical Engineering

University of Michigan,

1101 Beal Avenue, Ann Arbor, MI 48109, USA

[2]Center for Wireless Integrated MicroSensing and Systems (WIMS^2)

University of Michigan

Ann Arbor, MI 48109, USA

[3]Max Harry Weil Institute for Critical Care Research and Innovation

University of Michigan, Ann Arbor, MI 48109, USA

[4]Department of Electrical Engineering and Computer Science

University of Michigan

Ann Arbor, MI 48109, USA

*Corresponding author: xsfan@umich.edu

Section S1. Glossary of abbreviations and symbols

Abbreviation or symbols	Description
RT	Retention time of a compound, which is equal to the x-coordinate of the apex of the corresponding peak in a chromatogram.
ΔRT	The retention time deviation of the same compound in the chromatograms of different runs.
RTT	Retention time trajectory, which is made up of discrete points of retention time values of a list of compounds in a measured chromatogram.
RTT_{lib}	Retention time trajectory in a pre-characterized chromatogram installed in the library.
RTT_{sample}	Retention time trajectory for the chromatogram obtained from a sample under test, which is formed by pairing the detected peaks with a subset of target compounds.
N_{tgt}	Number of target analytes in a pre-determined list.
N_{sample}	Number of detected peaks in the chromatogram of the sample under test, with the added internal standards excluded.
N_{inf}	Number of interferents in the actual sample under test.
N_{std}	Number of internal standard compounds.
n_{lib}	Number of pre-characterized RTTs (RTT_{lib}s) in the library.
n_{sample}	Number of all possible RTTs generated from the chromatogram of the sample under test.
SSR	Sum of squared residuals of RTs from the same compounds between one RTT_{sample} and one RTT_{lib}. A residual is the difference between the RT (treated as observed value) of a peak in one sample chromatogram and the RT of paired compound in one RTT_{lib} (treated as predicted value).
MSR	Mean squared residual, which is the *SSR* normalized by the total of number of paired compounds.

Section S2. Algorithm validation tests generation

Depending on the number of target compounds, the sample for targeted analysis can be classified as: (1) all target compounds are present, (2) a subset of target compounds are present, and (3) a subset of target compounds as well as interferents are present. In the first scenario the peak identification can be easily achieved as the peaks in the sample chromatogram and in one pre-characterized chromatogram can be paired respectively (and therefore identified) based on the elution order. Peak identifications for samples of the other two scenarios are much more challenging, as the peaks can be paired with any subset of the target compounds. Therefore, in this work, we only discuss and show the details of 11 validation test results that fall under (2) and (3).

In our RTT matching method, the only inputs are the retention times. Therefore, one chromatogram, which is made up of a large 2D array of detection signal intensity vs. time, can be simplified as a list of retention times (Figure S7A). As the peak area and profile do not contribute to the peak identification, multiple algorithm validation tests can simply be generated from an actual chromatogram, which includes all target compounds and internal standards. This can be accomplished by selecting various subsets of the target compounds and adding hypothetical peak positions (*i.e.*, interferents), if needed.

In this work, a total of $3 \times \sum_{i=1}^{20}[\mathrm{C}(20, i)] = 3.15 \times 10^5$ validation tests were generated out of $\mathrm{Chrom}_{7\text{-}9}$, which cover *all* subsets of the 20 target compounds. Peak identifications in all validation tests achieved 100% accuracy. Particularly, 11 validation tests were selected and discussed in detail to represent samples of various compositions. Tests 1-3 (Table 1A) were generated from Chrom_7; Tests 4-9 (Tables 1B and S2) were generated from Chrom_8. Tests 10 and 11 (Table S3) were generated from Chrom_9. Peaks used in Tests 5-8 are exemplified in Figures S7B-E, respectively.

Section S3. Sample chromatogram reconstruction for COW aligning

Correlation optimized warping (COW) algorithm[1,2] is commonly used for chromatogram aligning. In order to compare the peak identification performance of COW aligning with RTT matching, a sample chromatogram (*i.e.*, $Chrom_{sample}$) is reconstructed out of $Chrom_8$, where only peaks listed in the validation test (plus two internal standards) are kept and the rest are replaced with the baseline. Test 5 (a subset of target compounds) and 7 (a subset of target compounds plus one internal standard), which have been validated with the RTT matching approach, are also used to evaluate peak identification with COW aligning.

In this work, the following steps for $Chrom_{sample}$ reconstruction were adopted. First, the open-source adaptive iterative reweighted Penalized Least Squares (airPLS) algorithm[3] was used to correct baseline drifting, and the baseline signal was numerically centered around zero. Second, the signal was smoothed via locally weighted scatterplot smoothing (LOWESS)[4] to further improve the signal-to-noise (S/N) ratio in $Chrom_8$. Next, the chromatogram curve was scanned for peak detection, where peak apex positions, peak heights and endpoints were extracted[5]. Each peak profile was fitted by an exponentially modified Gaussian (EMG) equation[6]. Finally, EMG expressions for all target compounds peaks listed in the validation tests (Tests 5 and 7), as well as two internal standards, were added to form a chromatogram ($Chrom_{sample}$) as the input for COW aligning. An artificial EMG expression was adopted to generate the interferent peak in Test 7. The $Chrom_{sample}$s for Tests 5 and 7 are plotted in Figure S8 together with $Chrom_8$.

During COW aligning, $Chrom_1$, where all target compounds and internal standards are present, was treated as the reference chromatogram for sample chromatograms to align with. Peak identification can be achieved only when the peaks of identical compounds are well aligned between $Chrom_1$ and $Chrom_{sample}$. Multiple COW aligning with various tuning parameters (slack and correlation power) were conducted; the corresponding aligned $Chrom_{sample}$s are plotted in Figures S8B-E and S9B-E. The identification results based on the retention times of the aligned $Chrom_{sample}$ are summarized in Table S4.

Section S4. Fruit metabolomics datasets

Two publicly available fruit metabolomics datasets from the Metabolights repository (http://www.ebi.ac.uk/metabolights) with identifiers MTBLS99 and MTBLS85 were used to demonstrate the potential of RTT matching for complicated samples. The same datasets were also used previously for fast PTW algorithm validation[7].

The first dataset consists of 23 measurements of a pooled sample that was injected regularly as a quality control (QC) during LC apple extracts measurements. The QC chromatograms are plotted in Figure S10A. The first 7 chromatogram sets (plotted in red) were used for RTT hybridization and subsequent RTT library construction. The remaining 16 chromatograms (plotted in blue) were used to generate validation tests. 40 peaks, which are marked with orange circles, were treated as the target compounds. 2 peaks, which are marked with green triangles, were used as internal standards. The remaining peaks show inconsistent elution pattern due to co-elution of different degrees across the chromatograms. They were therefore excluded from the target compound list. Following the same test design in Section S2, we chose target compound subsets containing 40, 30, 20, 10, and 5 target compounds and generated a total of $16 \times [\mathrm{C}(40,40) + \mathrm{C}(40,30) + \mathrm{C}(40,20) + \mathrm{C}(40,10) + \mathrm{C}(40,5)] = 2.23 \times 10^{12}$ tests. All peaks in the validation tests were correctly identified with the RTT matching approach.

The second datasets are from 14-day LC measurements of carotenoids in grape samples. In the fast PTW work[7], the data was clustered into 14 components according to spectral characteristics. Chromatograms in Component 7, which are plotted in Figure S10B, were used for validation. Similarly, chromatograms in the first 5 days (plotted in red) were used for RTT hybridization and subsequent RTT library construction. The remaining 9 chromatograms (plotted in blue) were used to generate validation tests. 13 peaks (marked with orange circles) were chosen as target compounds, and one peak (marked with green triangle) was used as the internal standard. Following the same test design in Section S2, we used all the target compound subsets (ranging from single compounds to all 13 compounds) for a total of $9 \times \sum_{i=1}^{13}[\mathrm{C}(13, i)] = 73{,}719$ tests. All peaks in the validation tests were correctly identified with the RTT matching approach.

Section S5. Chromatogram aligning enabled by RTT matching

In the main text, we mainly discuss the concept of RTT matching and its application in peak identification for chromatographic analysis. For visualization purposes, the RTT matching approach is also useful for chromatography profile aligning, which may be assisted by peak reconstruction if needed.

Generally, most chromatogram profile aligning approaches involve selection or generation of a reference chromatogram. All other sample chromatograms are aligned with the reference chromatogram and peaks of the same compound are aligned to the RT in the reference chromatogram. Herein, any RTT_{lib} can be chosen as the reference to extract RTs of target compounds (and internal standards if present). The peaks in the sample chromatogram are first identified with RTT matching and each peak can be shifted to the corresponding RTs in the reference chromatogram.

To eliminate the influence of baseline drifting on the peak profile, the sample chromatogram can be reconstructed by fitting each peak with an EMG model after baseline removal, smoothing and peak detection. Methods for each step can be found in Section S3. The aligned sample chromatogram is obtained by summation of all shifted EMG expressions. Illustration of RTT matching based chromatogram aligning is provided in Figure S11, where $Chrom_1$ was used as the reference chromatogram and two sample chromatograms were generated out of $Chrom_8$. $Chrom_{sample1}$ was created through summation and reconstruction of all peaks in $Chrom_8$ via EMG, representing a sample with the same analyte composition as the reference chromatogram. Similarly, $Chrom_{sample2}$ was reconstructed only with the peaks listed in Test 5 (Table 1B), representing a sample with only a subset of peaks in the reference chromatogram. $Chrom_{sample1}$ and $Chrom_{sample2}$ are plotted in Figure S11A together with $Chrom_8$, showing that all peak profiles and retention times are well preserved. Aligned chromatograms are shown in Figure S11B and C, together with the reference chromatogram (*i.e.*, $Chrom_1$), demonstrating excellent alignment between peaks of the same compounds.

Because peak RTs are the only input for the peak aligning, the RTT matching based chromatogram aligning completely avoids misaligning resulting from disparities in peak size, peak profile, and baseline drift, which are challenging for many other chromatogram aligning approaches to handle.

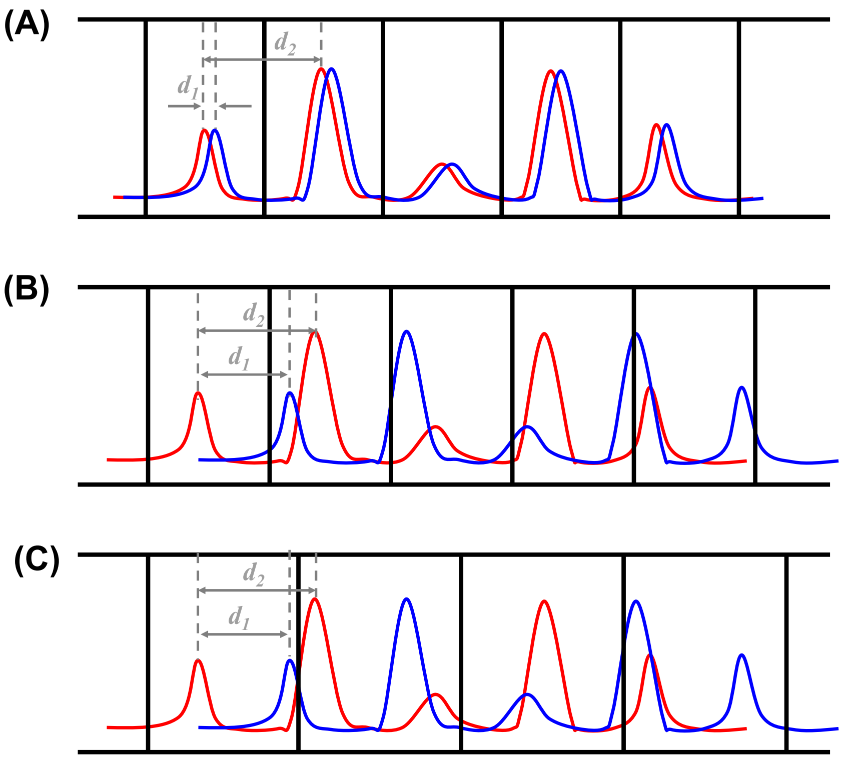

Figure S1. Conceptual illustration of the binning approach. **(A)** When drift of the same compound in two different chromatograms (red and blue), d_1, is much smaller than the distance between two neighboring peaks in the same chromatogram, d_2, peaks of the same compound can be well matched (that is, they are ended up in the same bin) via appropriate selection of the binning size. However, when d_1 and d_2 are comparable, as illustrated in **(B)** and **(C)**, peak mismatch occurs regardless of the binning size. **(B)** A narrow binning size incorrectly places the peaks from different compounds (*e.g.*, the first peak of the blue chromatogram and the second peak in the red chromatogram) into the same bin. **(C)** A wide binning size incorporates multiple peaks into the same bin (*e.g.*, the second and the third peak in the red chromatogram), which reduces analysis resolution.

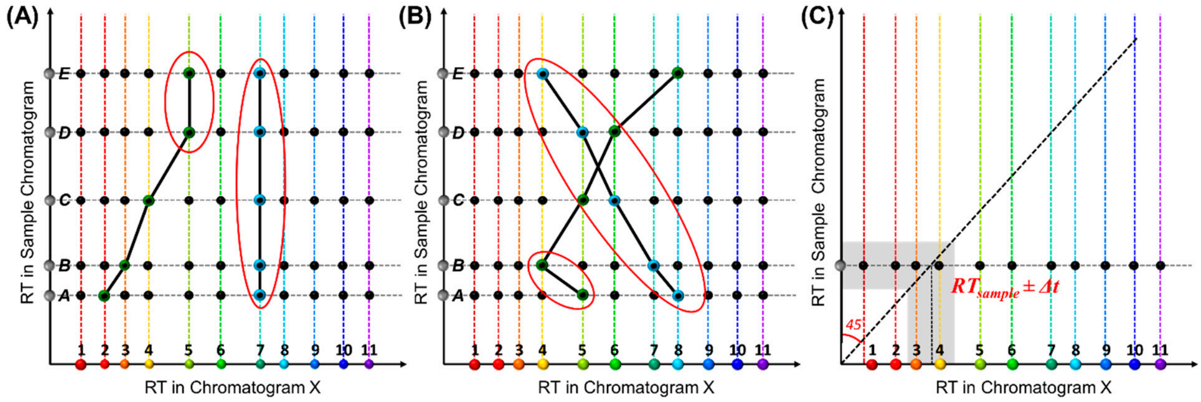

Figure S2. Rules to eliminate impossible RTT$_{sample}$s. **(A)** Two examples (circled in red) of impossible RTT$_{sample}$s, in which there is at least one vertical segment between two consecutive coordinates (black dots). A vertical segment means that one target compound is simultaneously assigned to multiple peaks in the chromatogram obtained from the sample under test. **(B)** Two examples (circled in red) of impossible RTT$_{sample}$s with an incorrect elution order, in which at least one segment between two consecutive coordinates (black dots) has a negative slope. **(C)** Only the coordinates (black dots) falling in the grey-shaded area need to be considered to expedite computation.

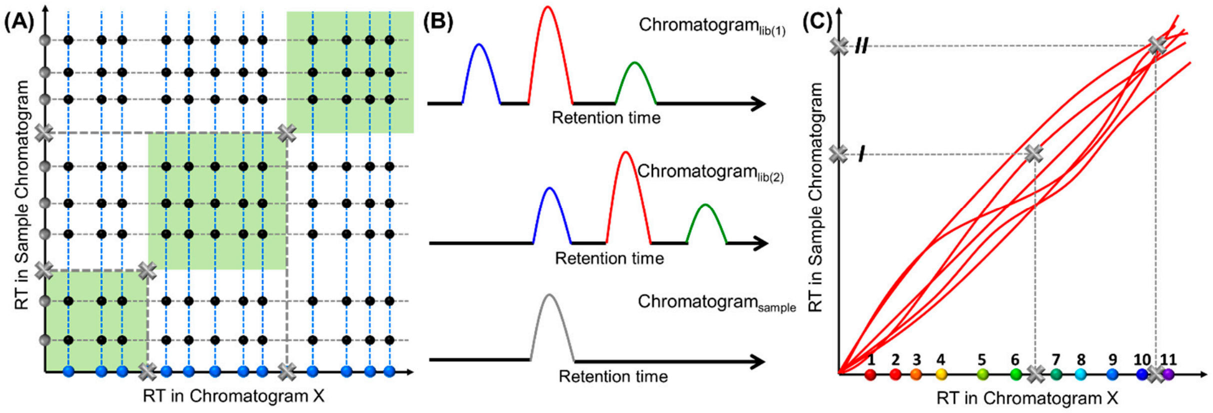

Figure S3. Use of internal standards. **(A)** Internal standards (grey crosses) divide the 2D diagram into multiple sub-sections (green regions). All possible RTT_{sample}s must go through the grey crosses. Therefore, only the black dots (coordinates) falling within these regions can be used to form possible RTT_{sample} candidates. **(B)** RT-based identification of a sample containing a single analyte is impossible, since the same RT value can also result from drifting neighboring peaks. In the examples presented here, the single peak in the sample chromatogram can be identified as the blue peak or red peak by the two chromatograms in the library. **(C)** Internal standards should be strategically positioned in the regions where variations in RTTs are more drastic. For example, Compound *I* is more effective than Compound *II* as an anchor for the trajectories because RTTs vary more significantly in the Compound *I* region than in the Compound *II* region.

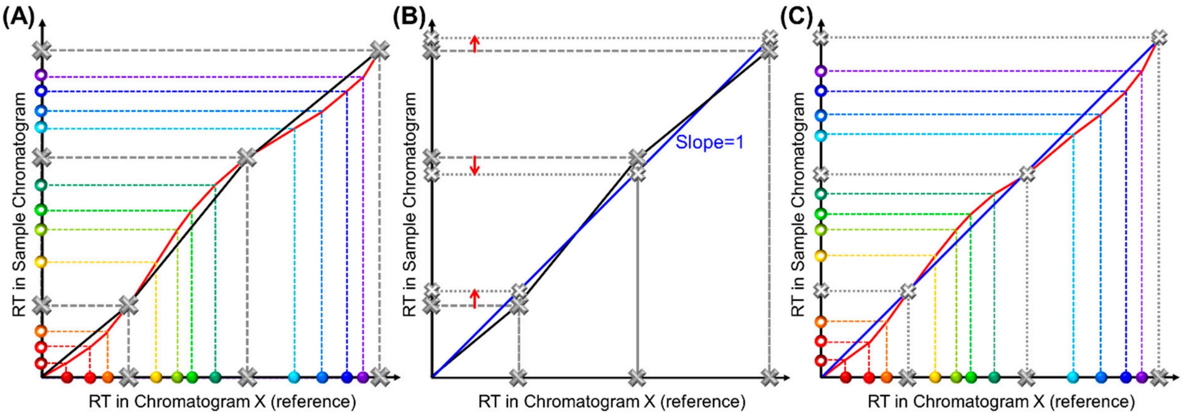

Figure S4. Conceptual illustration of peak aligning with the internal standard based linear stretching/compressing approach using the RTT 2D diagram. **(A)** Retention times in the sample chromatogram and those in Chromatogram X (*i.e.*, "reference chromatogram" used in the linear stretching/compressing approach) form the RTT_{sample} shown as the red curve. The first step of linear stretching/compressing is to connect the internal standards (solid gray crosses) using linear lines (marked as black straight lines). **(B)** The second step is to change the slope of those black lines to unity in order to match the retention times in the reference chromatogram (*i.e.*, Chromatogram X). Note that in the RTT 2D diagram, Chromatogram X is represented by a straight line with a slope of one (see the blue line, where the internal standards are marked as small hollow gray crosses). The red arrows point to the warping direction (stretching or compressing). Stretching/compressing is easily interpreted as a slope change (*i.e.*, the slopes in the three segments are all changed to unity) in the RTT 2D diagram. **(C)** A new RTT_{sample} (red curve) is formed from the original RTT_{sample} in (A) after taking into account the slope change in each segment described in (B). Apparently, the new RTT_{sample} still does not overlap with the blue curve (*i.e.*, the reference chromatogram). Consequently, there still exist differences between the retention times in the sample chromatogram and those in the reference chromatogram, which leads to misidentification of the peaks in the sample chromatogram.

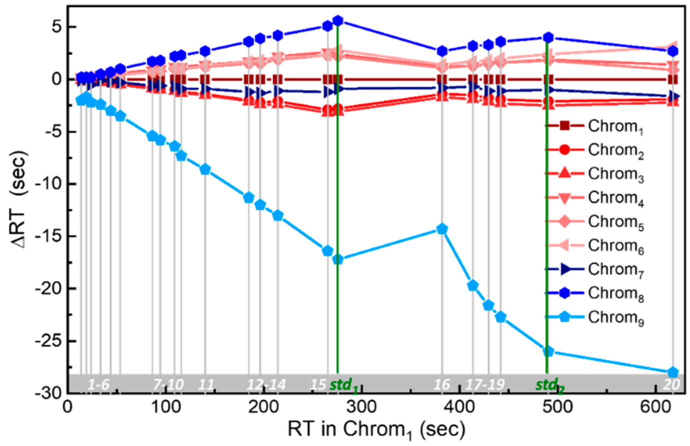

Figure S5. Retention time deviation (ΔRT) of $Chrom_{1\text{-}9}$ against the RT in $Chrom_1$. The X-axis represents the retention time obtained from $Chrom_1$. ΔRT along the Y-axis is obtained from the RT in $Chrom_{1\text{-}9}$ minus the RT of the same compound in $Chrom_1$. A positive (negative) deviation indicates that the corresponding compound elutes later (earlier) than in $Chrom_1$. ΔRT = 0 for all compounds in $Chrom_1$. ΔRT for all chromatograms, except for $Chrom_1$, are highly non-linear.

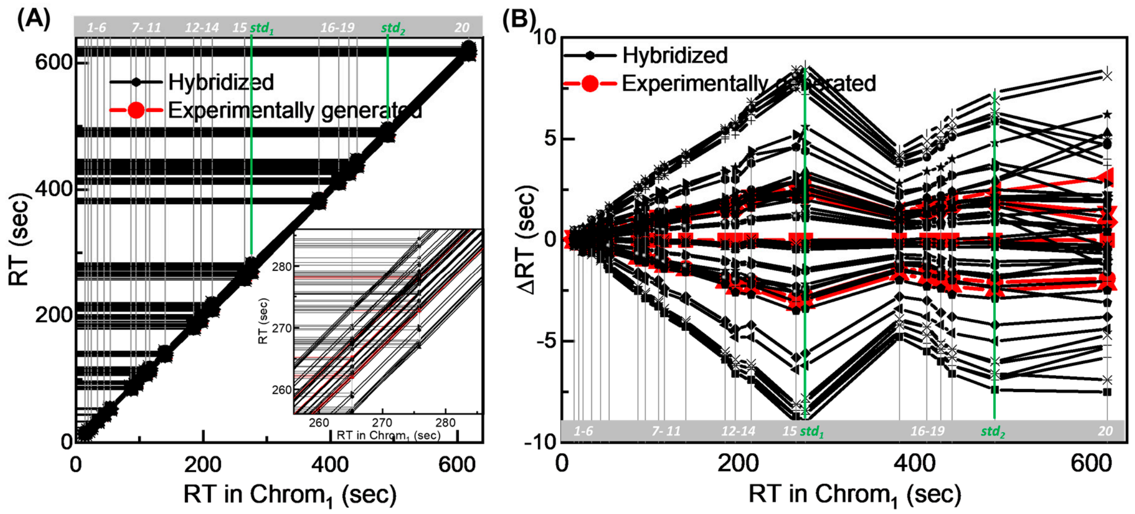

Figure S6. Demonstration of RTTlibs hybridization. **(A)** RTT library formed by experimentally generated RTTlibs (RTT1-6, red) and linearly hybridized RTTlibs (black) based on RTT1-6. **(B)** Corresponding retention time deviations (ΔRTs) against RTs in Chrom1.

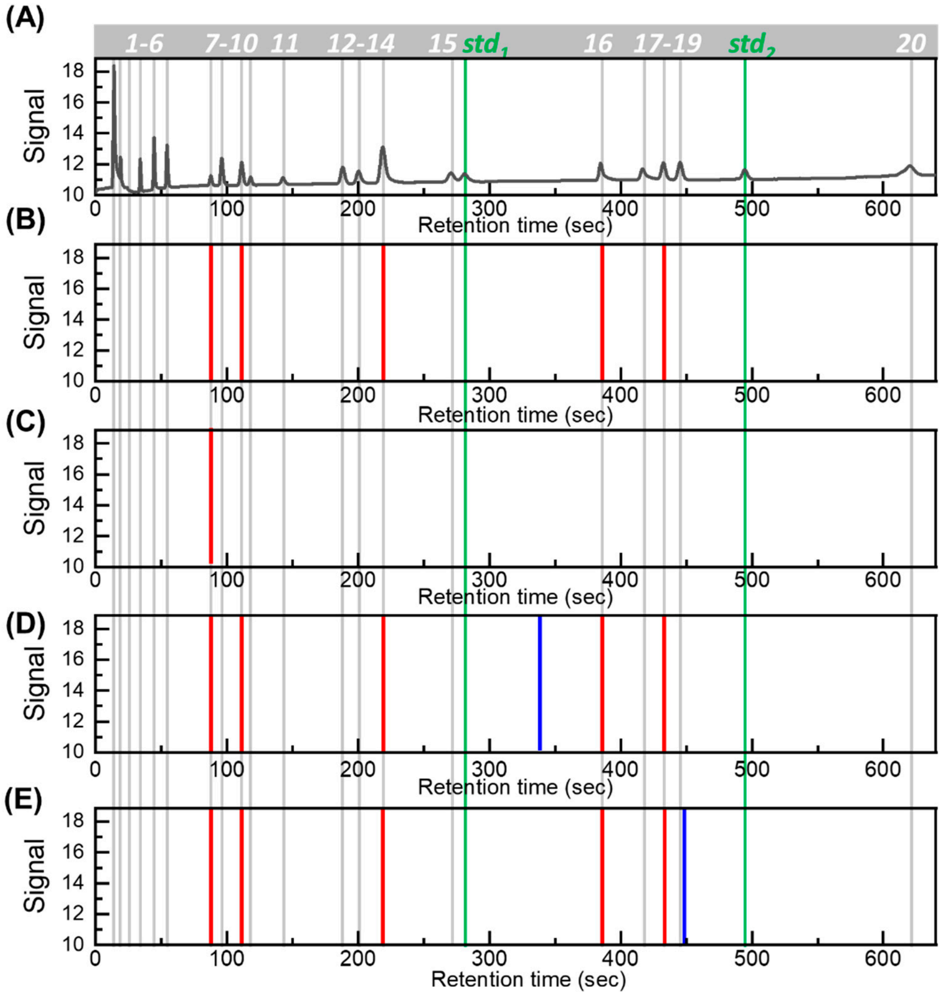

Figure S7. Illustration of algorithm validation tests design using Chrom8. **(A)** Experimentally generated Chrom8, where all target compounds and internal standards are present. Peak positions are marked with grey lines. Corresponding peak IDs are listed in the grey bar above. **(B-E)** Illustration of the peaks used in Tests 5-8 generated from Chrom8. The red lines mark the peaks involved in the validation tests. The green lines mark the positions of the two internal standards. The red and green peaks are included in the sample under test. The peaks marked in grey are not included in the sample under test. Artificially added interferents are marked in blue.

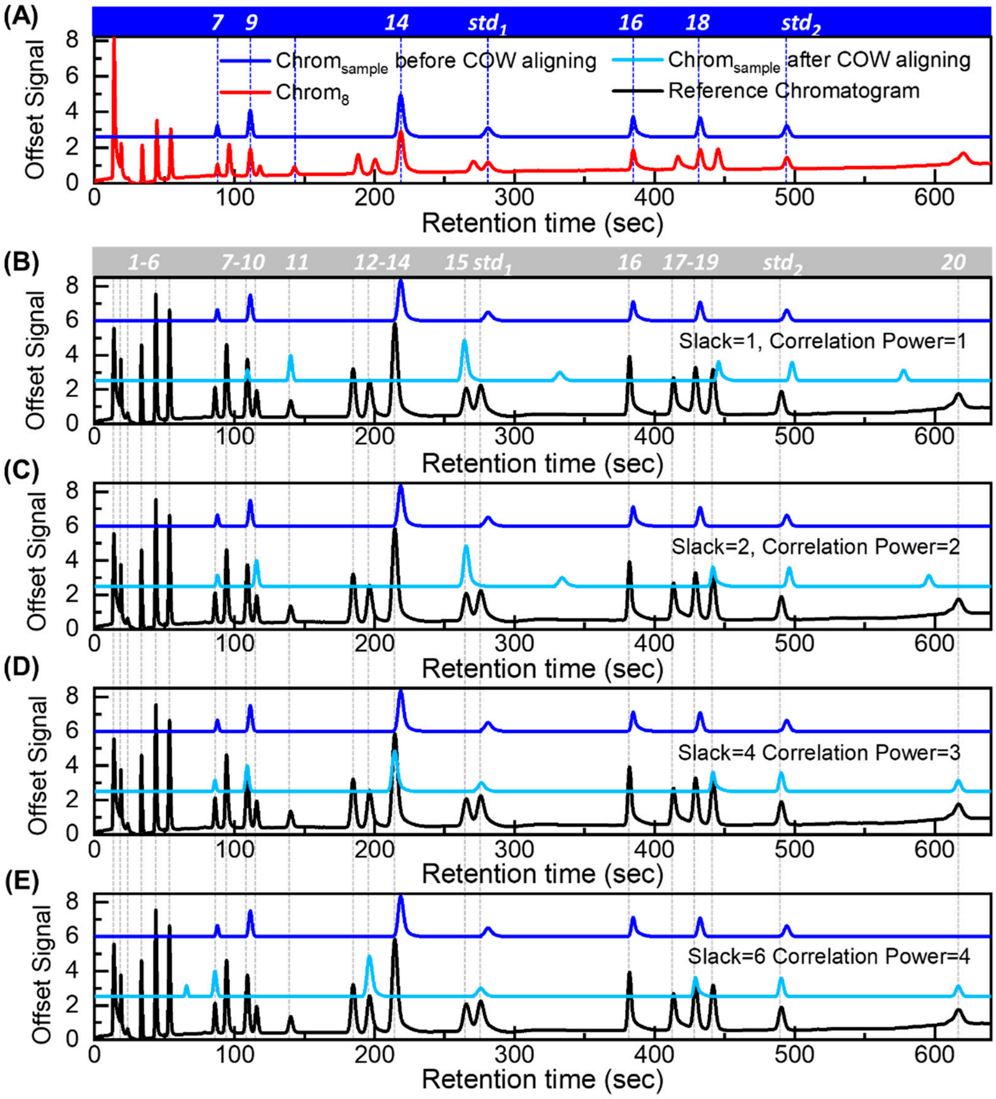

Figure S8. Peak identification using COW aligning with a sample containing a subset of target compounds (Test 5). **(A)** $Chrom_8$ and $Chrom_{sample}$ reconstructed from $Chrom_8$ with the peaks in Test 5. Peak retention times before aligning are listed in Table S2. Both peak positions and peak profiles are well preserved in $Chrom_{sample}$ after reconstruction. The corresponding peak IDs are marked in the top blue bar. $Chrom_{sample}$ is vertically shifted for clarity. **(B-E)** Reference chromatogram (*i.e.*, $Chrom_1$), unaligned $Chrom_{sample}$, and aligned $Chrom_{sample}$ using various COW tuning parameters (slack and correlation power). Peak positions and corresponding peak IDs are labelled in the top grey bar. Multiple peaks are aligned to incorrect peaks in the reference chromatogram, leading to

misidentification. Identification results are summarized in Table S4A. Unaligned $Chrom_{sample}$ and aligned $Chrom_{sample}$ are vertically shifted for clarity.

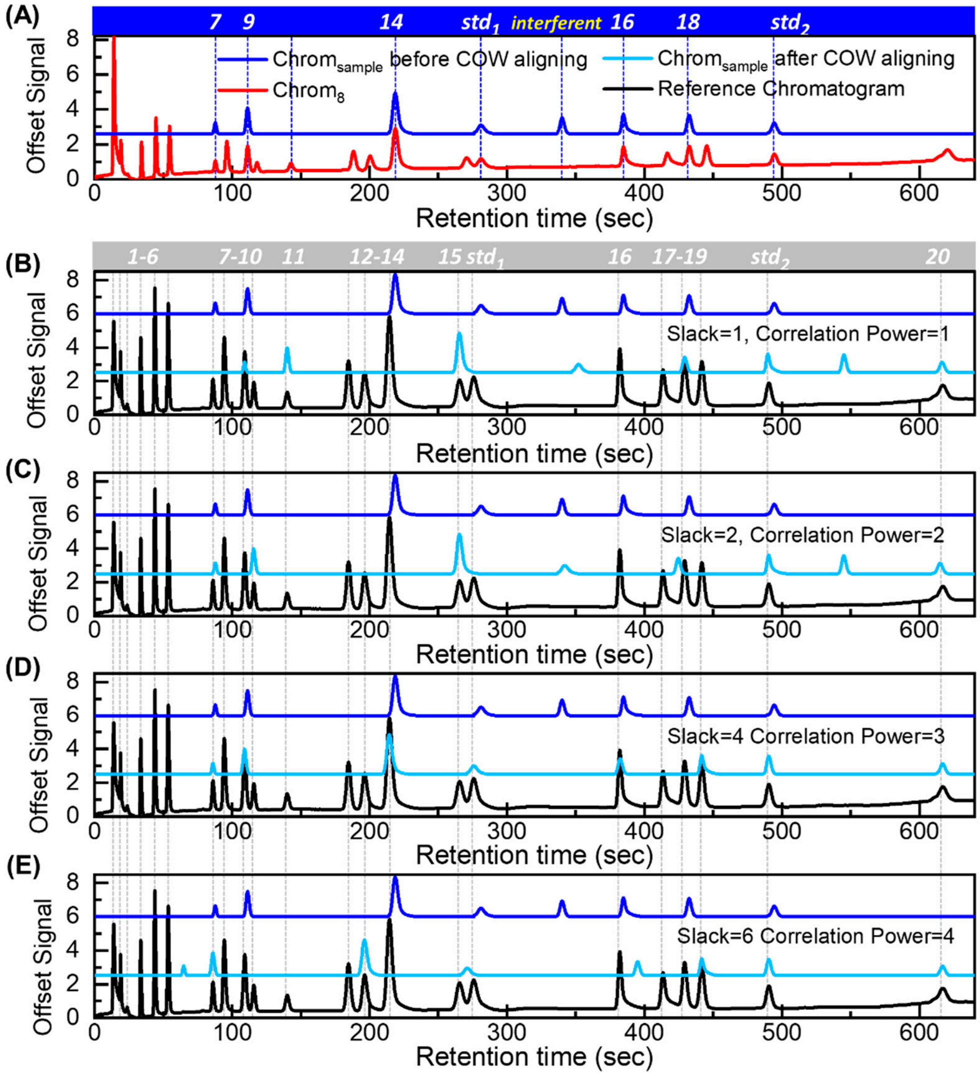

Figure S9. Peak identification using COW aligning with a sample containing a subset of target compounds plus one interferent (Test 7). **(A)** $Chrom_8$ and $Chrom_{sample}$ reconstructed from $Chrom_8$ with the target peaks in Test 7. One interferent peak is artificially added at 340 s with its peak profile generated by an EMG. Peak retention times before aligning are listed in Table S2. Both peak positions and peak profile are well preserved for the target compounds in $Chrom_{sample}$ after the reconstruction. The corresponding peak IDs are marked in the blue bar above. $Chrom_{sample}$ is vertically shifted for clarity. **(B-E)** The reference chromatogram (*i.e.*, $Chrom_1$), unaligned $Chrom_{sample}$, and aligned $Chrom_{sample}$ using various COW tuning parameters (slack and correlation power). Peak positions and corresponding peak IDs are labelled in the top grey bar. Multiple peaks are aligned to the incorrect peaks in the reference chromatogram, leading to misidentification. Identification results are summarized in Table S4B. Unaligned $Chrom_{sample}$ and aligned $Chrom_{sample}$ are vertically shifted for clarity.

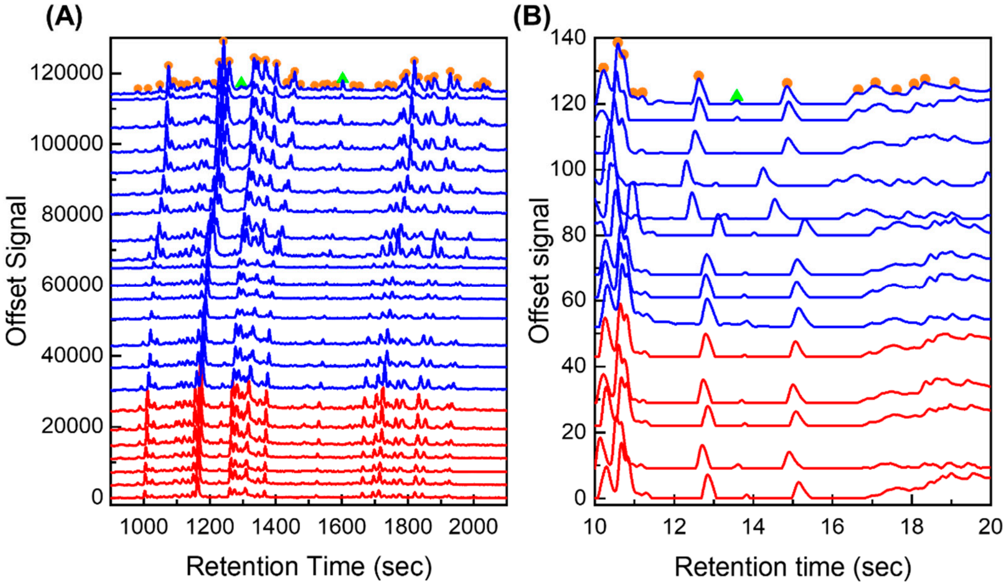

Figure S10. Fruit metabolomics chromatograms for RTT peak identification verification. Chromatograms plotted in red are used for RTT library construction and blue ones are used for verification tests design. The orange dots mark the peaks that are treated as target compounds. The green triangles mark the peaks used as internal standards. **(A)** A pooled sample used as QC during the apple extracts measurement. **(B)** Component 7 of carotenoids in grape samples. A detailed sample description can be found in Ref. 7.

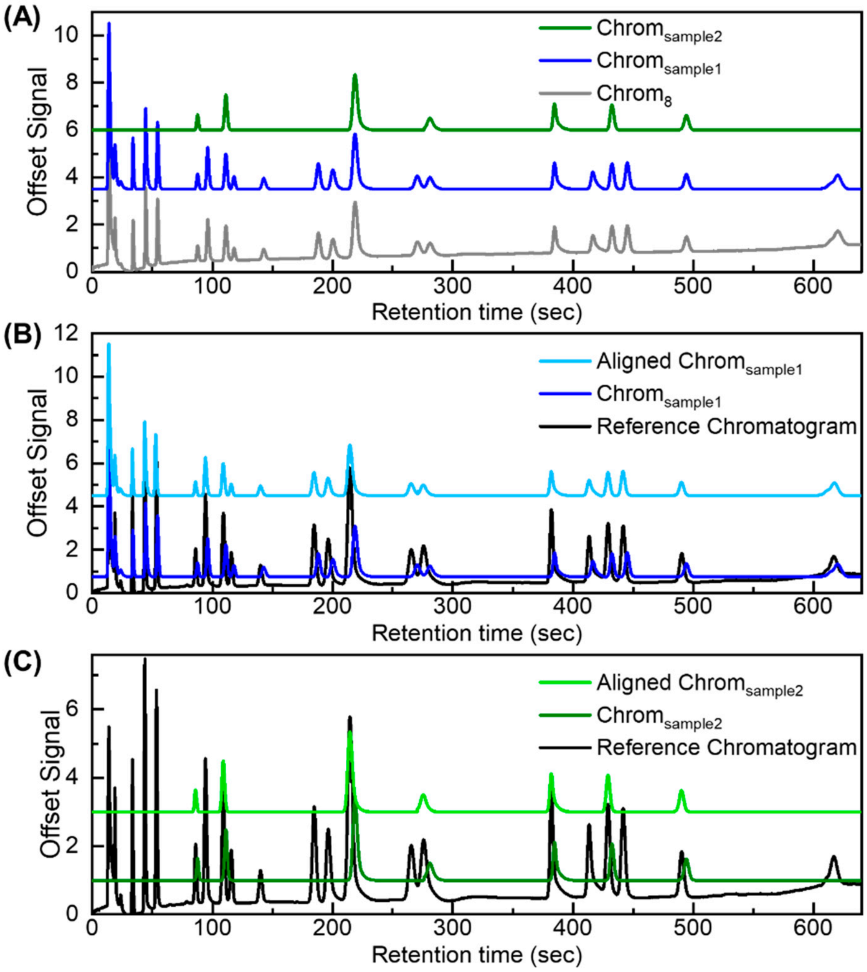

Figure S11. Chromatogram aligning enabled by RTT matching. **(A)** Two sample chromatograms ($\text{Chrom}_{\text{sample1}}$ and $\text{Chrom}_{\text{sample2}}$) generated from Chrom_{8}. $\text{Chrom}_{\text{sample1}}$ is reconstructed by fitting all peaks in Chrom_{8} with EMGs, whereas $\text{Chrom}_{\text{sample2}}$ only keeps the peaks listed in Test 5. Both retention times and peak profiles are well preserved for all target compounds in $\text{Chrom}_{\text{sample}}$ after reconstruction. **(B)** and **(C)** Sample chromatograms ($\text{Chrom}_{\text{sample1}}$ and $\text{Chrom}_{\text{sample2}}$) before and after the alignment. Chrom_{1} is used as the reference chromatogram to extract peak RTs for alignment.

Retention Time (sec)	Compound ID	Compound Name
13.9	1	Unknown
19	2	1,1-Dichloroethene
23.7	3	Unknown
33.6	4	*cis*-1,2-Dichloroethene
43.9	5	Benzene
53.6	6	Trichloroethylene
86.2	7	*cis*-1,3-Dichloropropene
94.4	8	Toluene
109.2	9	Tetrachloroethylene
115.9	10	*trans*-1,3-Dichloropropene
140.2	11	1,2-Dibromoethane
184.8	12	Chlorobenzene
196.5	13	Ethylbenzene
214.4	14	*m,p*-Xylene
265.2	15	*o*-Xylene
275.8	std_1	Styrene
382.1	16	1,3,5-Trimethylbenzene
413.4	17	1,2,4-Trimethylbenzene
429.3	18	1,3-Dichlorobenzene
441.8	19	1,4-Dichlorobenzene
490.5	std_2	1,2-Dichlorobenzene
617.6	20	Hexachloro-1,3-Butadiene

Table S1. Peak retention times, assigned compound IDs, and compound names in $Chrom_1$. The same elution order holds for all chromatograms discussed in this work.

Test data generated from Chrom$_8$

Test 7

Retention time (sec)			87.9	111.4	218.6	340	384.8	432.6
**Compound ID**			7	9	14	Interferent	16	18
**Ranking**	**MSR**	**Accuracy**	**Individual peak identification result**					
1st	2.99	100%	7	9	14	Interferent	16	18
2nd	3.74	100%	7	9	14	Interferent	16	18
3rd	4.28	100%	7	9	14	Interferent	16	18
4th	7.14	83.3%	7	10*	14	Interferent	16	18

Test 8

Retention time (sec)			87.9	111.4	218.6	384.8	432.6	449
**Compound ID**			7	9	14	16	18	Interferent
**Ranking**	**MSR**	**Accuracy**	**Individual peak identification result**					
1st	2.99	100%	7	9	14	16	18	Interferent
2nd	3.74	100%	7	9	14	16	18	Interferent
3rd	4.11	83.3%	7	9	14	16	18	19*
4th	4.28	100%	7	9	14	16	18	Interferent

Test 9

Retention time (sec)			34.1	44.6	62	96.2	111.4	142.9	188.4	218.6	384.8	395	432.6
**Compound ID**			4	5	interferent	8	9	11	12	14	16	interferent	18
**Ranking**	**MSR**	**Accuracy**	**Individual peak identification result**										
1st	2.42	100%	4	5	interferent	8	9	11	12	14	16	interferent	18
2nd	2.86	100%	4	5	interferent	8	9	11	12	14	16	interferent	18
3rd	3.34	100%	4	5	interferent	8	9	11	12	14	16	interferent	18
4th	5.06	90.9%	4	5	interferent	8	10*	11	12	14	16	interferent	18

Table S2. Algorithm validation tests and the corresponding peak identification results. The sample under test consists of both target compounds and interferents. Retention times for target compounds are generated from Chrom$_8$. An interferent at 340 s in Test 7 and at 449 s in Test 8 are added artificially. In Test 9, two interferents are artificially added at 62 s and 385 s. An asterisk "*" denotes peak misidentification.

Test data generated from $Chrom_9$

Test 10

Retention time (sec)			11.92	21.52	31.2	40.8	50	80.8	108.8	131.6	173.6	248.8	393.6
**Compound ID**			1	3	4	5	6	7	10	11	12	15	17
**Ranking**	**MSR**	**Accuracy**	**Individual peak identification result (with experimentally generated RTT_{libs} only)**										
1st	98.99	81.8%	1	3	4	5	6	7	9*	11	12	15	16*
2nd	99.13	72.7%	1	2*	4	5	6	7	9*	11	12	15	16*
3rd	101.48	81.8%	1	3	4	5	6	7	9*	11	12	15	16*
4th	101.66	72.7%	1	2*	4	5	6	7	9*	11	12	15	16*
**Ranking**	**MSR**	**Accuracy**	**Individual peak identification result (with both experimentally generated and hybridized RTT_{libs})**										
1st	1.13	100%	1	3	4	5	6	7	10	11	12	15	17
2nd	2.55	90.9%	1	2*	4	5	6	7	10	11	12	15	17
3rd	3.16	90.9%	1	3	4	5	6	7	9*	11	12	15	17
4th	3.29	90.9%	2*	3	4	5	6	7	10	11	12	15	17

Test 11

Retention time (sec)			31.2	50.1	88.6	108.6	173.5	248.8	419.1
**Compound ID**			4	6	8	10	12	15	19
**Ranking**	**MSR**	**Accuracy**	**Individual peak identification result (with experimentally generated RTT_{libs} only)**						
1st	121.41	57.1%	4	6	7*	9*	12	15	17*
2nd	122.28	57.1%	4	6	7*	9*	12	15	18*
3rd	122.76	71.4%	4	6	8	9*	12	15	17*
4th	123.63	71.4%	4	6	8	9*	12	15	18*
**Ranking**	**MSR**	**Accuracy**	**Individual peak identification result (with both experimentally generated and hybridized RTT_{libs})**						
1st	1.64	100%	4	6	8	10	12	15	19
2nd	4.58	85.7%	4	6	8	9*	12	15	19
3rd	7.38	85.7%	4	6	7*	10	12	15	19
4th	7.40	100%	4	6	8	10	12	15	19

Table S3. Algorithm validation tests and the corresponding peak identification results when a sample chromatogram has severe RT drift issues. Retention times for compounds in Test 9 and Test 10 were generated from $Chrom_9$, which drift much more seriously than $Chrom_{7\text{-}8}$ (see Figure S5). In each test, severe peak misidentification occurred when only the experimentally generated RTT_{libs} (*i.e.*, $Chrom_{1\text{-}6}$) were used. In contrast, when the RTT_{libs} generated by the hybridization method were added, our approach could identify the peaks with 100% accuracy (at least for the top result with the smallest *MSR*). An asterisk "*" denotes peak misidentification.

RT in *Test 5* (sec)	87.9	111.4	218.6	281.4	384.8	432.6	494.5
**Compound ID**	7	9	14	std_1	16	18	std_2
**Individual peak identification result w/ COW aligning (slack=1, correlation power=1), Accuracy=0**							
**RT after aligning (sec)**	109.2	140.2	265.2	449.5	497.1	497.1	575.7
**Peak identification**	9*	11*	15*	Interferent*	Interferent*	Interferent*	Interferent*
**Individual peak identification result w/ COW aligning (slack=2, correlation power=2), Accuracy=0**							
**RT after aligning (sec)**	88.2	115.9	265.2	345.8	441.8	499.2	597.3
**Peak identification**	Interferent*	10	15	Interferent*	19*	Interferent*	Interferent*
**Individual peak identification w/ COW aligning (slack=4, correlation power=3), Accuracy=57.1%**							
**RT after aligning (sec)**	86.2	109.2	214.4	281.4	441.8	490.5	617.6
**Peak identification**	7	9	14	std_1	19*	std_2*	20*
**Individual peak identification result w/ COW aligning (slack=6, correlation power=4), Accuracy=28.6%**							
**RT after aligning (sec)**	65.1	86.2	196.5	281.4	429.3	490.5	617.6
**Peak identification**	Interferent*	7*	13*	std_1	18	std_2*	20*

(A)

(B)

RT in *Test 7* (sec)	87.9	111.4	218.6	281.4	340	384.8	432.6	494.5
**Compound ID**	7	9	14	std_1	Interferent	16	18	std_2
**Individual peak identification result w/ COW aligning (slack=1, correlation power=1), Accuracy=0**								
**RT after aligning (sec)**	109.2	140.2	265.2	354.1	429.3	490.5	545.4	617.6
**Peak identification**	9*	11*	15*	Interferent*	18*	std_2	Interferent*	20
**Individual peak identification result w/COW aligning (slack=2, correlation power=2), Accuracy=0**								
**RT after aligning (sec)**	88.5	115.9	265.2	341.9	425.1	490.5	545	617.6
**Peak identification**	Interferent*	10*	15*	Interferent*	Interferent	std_2*	Interferent*	20*
**Individual peak identification result w/ COW aligning (slack=4, correlation power=3), Accuracy=50%**								
**RT after aligning (sec)**	86.2	109.2	214.4	275.8	382.1	441.8	490.5	617.6
**Peak identification**	7	9	14	std_1	16*	19*	std_2*	20*
**Individual peak identification result w/ COW aligning (slack=6, correlation power=4), Accuracy=12.5%**								

RT after aligning (sec)	64.7	86.2	196.5	271.8	394.7	441.8	490.5	617.6
**Peak identification**	Interferent *	7*	13*	Interferent *	Interferent	19*	std_2*	20*

Table S4. Peak identification performance comparison with COW using Test 5 in Table **(A)** and Test 7 in Table **(B)**. $Chrom_1$ was treated as the reference chromatogram for other chromatograms (*i.e.*, sample chromatograms) to align with. The two sample chromatograms (*i.e.*, $Chrom_{sample}$s), with or without interferent, were generated from $Chrom_8$, as shown in Figures S8A and S9A. RTs and corresponding compound IDs are listed in the first two rows in each table. Peak identifications and RTs after COW with various parameters are summarized in the remaining rows. An asterisk "*" denotes peak misidentification.

Test 5							
**RT before aligning (sec)**	87.9	111.4	218.6	281.4	384.8	432.6	494.5
**Compound ID**	7	9	14	std_1	16	18	std_2
**Individual peak identification result with linear warping<br>Same internal standards (std_1 and std_2) are adopted, Accuracy=28.6%**							
**RT after aligning (sec)**	86.15	109.18	214.3	275.8	380.0	428.1	490.5
**Peak identification**	Interferent*	Interferent*	Interferent*	std_1	Interferent*	Interferent*	std_2

Test 7								
**RT before aligning (sec)**	87.9	111.4	218.6	281.4	340	384.8	432.6	494.5
**Compound ID**	7	9	14	std_1	Interferent	16	18	std_2
**Individual peak identification result with linear warping<br>Same internal standards (std_1 and std_2) are adopted, Accuracy=25%**								
**RT after aligning (sec)**	86.315	109.18	214.3	275.8	334.84	380.0	428.1	490.5
**Peak identification**	Interferent*	Interferent*	Interferent*	std_1	Interferent*	Interferent*	Interferent*	std_2

Table S5. Peak identification performance comparison with internal standard based linear warping using Tests 5 and 7. The same internal standards (*i.e.*, std_1 and std_2) were used for both tests. $Chrom_1$ was treated as the reference chromatogram. The sample chromatogram peak list (*i.e.*, $Chrom_{sample}$) was generated from $Chrom_8$. Corresponding compound IDs are listed in the first two rows of each table. Peak identifications and RTs after linear warping are summarized in the remaining rows. An asterisk "*" denotes peak misidentification.

(B)

Chrom 8	Test 5	CompoundID	7	9	14	std1	16	18	std2
		**RT**	87.9	111.4	218.6	281.0	384.6	432.4	494.3
		**Peak height**	0.64	1.49	2.34	0.50	1.11	1.07	0.62
		**Warped RT, Order=2**	59.10	86.20	206.67	274.11	381.95	429.96	490.50
		**Warped RT, Order=3**	239.66	109.20	28.11	196.50	429.30	386.54	68.11

Chrom 8	Test 7	CompoundID	7	9	14	std1	interferent	16	18	std2
		**RT**	87.9	111.4	218.6	281.0	340.0	384.6	432.4	494.3
		**Peak height**	0.64	1.49	2.34	0.50	0.92	1.11	1.07	0.62
		**Warped RT, Order=2**	59.04	86.20	206.86	274.33	336.36	382.10	430.03	490.41

(A)

**Compound ID**	**Reference**		**Chrom8**				**Chrom9**			
	**RT**	**Peak height**	**RT**	**Peak height**	**Warped RT, Order=2**	**Warped RT, Order=3**	**RT**	**Peak height**	**Warped RT, Order=2**	**Warped RT, Order=3**
**1**	13.9	17.65	14.1	18.37	14.14	14.42	11.9	2.36	12.47	11.26
**2**	19	15.86	19.2	12.44	19.11	19.34	17.3	7.06	18.27	17.17
**3**	23.7	12.73	23.9	10.50	23.69	23.88	21.5	2.52	22.78	21.76
**4**	33.6	16.69	34.1	12.33	33.63	33.73	31.2	7.43	33.19	32.35
**5**	43.9	19.63	44.6	13.72	43.87	43.90	40.9	9.08	43.59	42.91
**6**	53.6	18.72	54.6	13.24	53.63	53.60	50.1	8.49	53.44	52.90
**7**	86.2	14.21	87.9	11.26	86.20	86.02	80.8	3.90	86.26	86.07
**8**	94.4	16.72	96.2	12.38	94.33	94.13	88.6	6.45	94.58	94.46
**9**	109.2	15.85	111.4	12.11	109.23	109.00	102.8	5.20	109.72	109.69
**10**	115.9	14.04	118.2	11.17	115.90	115.66	108.6	3.87	115.90	115.90
**11**	140.2	13.45	142.9	11.12	140.16	139.93	131.6	2.74	140.36	140.45
**12**	184.8	15.31	188.4	11.80	184.98	184.83	173.5	4.84	184.80	184.89
**13**	196.5	14.65	200.4	11.54	196.82	196.71	184.5	4.29	196.44	196.50
**14**	214.4	17.94	218.6	13.11	214.81	214.76	201.4	6.84	214.29	214.30
**15**	265.2	14.18	270.3	11.43	266.04	266.22	248.8	3.80	264.22	264.02
**std1**	275.8	14.35	281.4	11.38	277.06	277.30	258.6	3.95	274.52	274.26
**16**	382.1	16.02	384.8	12.06	380.20	380.88	367.8	6.01	388.61	387.89
**17**	413.4	14.79	416.6	11.70	412.08	412.84	393.7	4.41	415.50	414.77
**18**	429.3	15.38	432.6	12.09	428.15	428.93	407.7	4.56	430.00	429.30
**19**	441.8	15.26	445.4	12.11	441.02	441.80	419.1	4.36	441.80	441.13
**std2**	490.5	14.00	494.5	11.64	490.50	491.20	464.5	3.36	488.66	488.31
**20**	617.6	13.86	620.3	11.89	618.09	617.60	588.1	2.76	615.21	617.60

		Warped RT, Order=3	121.10	86.20	23.70	52.08	115.90	184.85	275.80	415.47

Chrom 9	Test 10	CompoundID	1	3	4	5	6	7	10	11	12	15	std1	17	std2
		RT	11.9	21.5	31.2	40.9	50.1	80.8	108.6	131.6	173.5	248.8	258.6	393.7	464.5
		Peak height	2.36	2.52	7.43	9.08	8.49	3.90	3.87	2.74	4.84	3.80	3.95	4.41	3.36
		Warped RT, Order=2	-0.46	10.85	22.25	33.60	44.33	79.88	111.73	137.83	184.80	267.35	277.91	419.43	490.50
		Warped RT, Order=3	273.93	181.38	109.20	56.76	23.70	11.71	96.76	206.06	416.46	441.80	378.43	-3416.86	-8571.08

Chrom 9	Test 11	CompoundID	4	6	8	10	12	15	std1	19	std2
		RT	31.2	50.1	88.6	108.6	173.5	248.8	258.6	419.1	464.5
		Peak height	7.43	8.49	6.45	3.87	4.84	3.80	3.95	4.36	3.36
		Warped RT, Order=2	21.06	43.90	89.49	112.67	185.59	265.72	275.80	429.28	468.75
		Warped RT, Order=3	19.00	43.61	91.84	115.90	189.44	266.42	275.80	410.06	441.80

Table S6. Peak identification performance comparison with fast PTW. The full peak list in Chrom$_1$ is treated as a reference for alignment. PTW warping functions are applied with order 2 and 3. All retention times in the table are provided in seconds. Table **(A)** summarizes the full peak lists (retention times and peak heights) in Chrom$_1$, Chrom$_8$, and Chrom$_9$, as well as warped retention times. The retention time of each compound after warping is close to that in the reference (with a difference of a fraction of a second to a few seconds). Table **(B)** summarizes the subset peak lists in Tests 5 and 7 (generated out of Chrom$_8$), and Tests 10 and 11 (generated out of Chrom$_9$), as well as warped retention times. The retention time of a compound after warping deviates significantly from that in the reference (by a fraction of a second to hundreds of seconds), suggesting that the fast PTW may not be able to handle the situation when only a subset of the target compounds is present in the sample.

References

(1) Nielsen, N. P. V.; Carstensen, J. M.; Smedsgaard, J. Aligning of Single and Multiple Wavelength Chromatographic Profiles for Chemometric Data Analysis Using Correlation Optimised Warping. *J. Chromatogr. A* **1998**, *805* (1–2), 17–35.

(2) Tomasi, G.; Van Den Berg, F.; Andersson, C. Correlation Optimized Warping and Dynamic Time Warping as Preprocessing Methods for Chromatographic Data. *J. Chemom.* **2004**, *18* (5), 231–241.

(3) Zhang, Z. M.; Chen, S.; Liang, Y. Z. Baseline Correction Using Adaptive Iteratively Reweighted Penalized Least Squares. *Analyst* **2010**, *135* (5), 1138–1146.

(4) Cleveland, W. S.; Devlin, S. J. Locally Weighted Regression: An Approach to Regression Analysis by Local Fitting. *J. Am. Stat. Assoc.* **1988**, *83* (403), 596–610.

(5) Morris, J. S.; Coombes, K. R.; Koomen, J.; Baggerly, K. A.; Kobayashi, R. Feature Extraction and Quantification for Mass Spectrometry in Biomedical Applications Using the Mean Spectrum. *Bioinformatics* **2005**, *21* (9), 1764–1775.

(6) Lee, J.; Zhou, M.; Zhu, H.; Nidetz, R.; Kurabayashi, K.; Fan, X. Fully Automated Portable Comprehensive 2-Dimensional Gas Chromatography Device. *Anal. Chem.* **2016**, *88* (20), 10266–10274.

(7) Wehrens, R.; Bloemberg, T. G.; Eilers, P. H. C. Fast Parametric Time Warping of Peak Lists. *Bioinformatics* **2015**.
